# The Hemagglutinin of Influenza A Virus Induces Ferroptosis to Facilitate Viral Replication

**DOI:** 10.1002/advs.202404365

**Published:** 2024-08-19

**Authors:** Aotian Ouyang, Tong Chen, Yi Feng, Jiahui Zou, Shaoyu Tu, Meijun Jiang, Huimin Sun, Hongbo Zhou

**Affiliations:** ^1^ National Key Laboratory of Agricultural Microbiology College of Veterinary Medicine Huazhong Agricultural University Wuhan Hubei 430070 China; ^2^ Frontiers Science Center for Animal Breeding and Sustainable Production Wuhan Hubei 430070 China; ^3^ Hubei Hongshan Laboratory Wuhan Hubei 430070 China; ^4^ Key Laboratory of Preventive Veterinary Medicine in Hubei Province the Cooperative Innovation Center for Sustainable Pig Production Wuhan Hubei 430070 China

**Keywords:** autophagy, ferroptosis, influenza a virus, innate immunity, mitochondrial antiviral signaling protein (MAVS)

## Abstract

Ferroptosis is a novel form of cell death caused by the accumulation of lipid peroxides in an iron‐dependent manner. However, the precise mechanism underlying the exploitation of ferroptosis by influenza A viruses (IAV) remains unclear. The results demonstrate that IAV promotes its own replication through ferritinophagy by sensitizing cells to ferroptosis, with hemagglutinin identified as a key trigger in this process. Hemagglutinin interacts with autophagic receptors nuclear receptor coactivator 4 (NCOA4) and tax1‐binding protein 1 (TAX1BP1), facilitating the formation of ferritin‐NCOA4 condensates and inducing ferritinophagy. Further investigation shows that hemagglutinin‐induced ferritinophagy causes cellular lipid peroxidation, inhibits aggregation of mitochondrial antiviral signaling protein (MAVS), and suppresses the type I interferon response, thereby contributing to viral replication. Collectively, a novel mechanism by which IAV hemagglutinin induces ferritinophagy resulting in cellular lipid peroxidation, consequently impairing MAVS‐mediated antiviral immunity, is revealed.

## Graphical abstract

The precise relationship between influenza A virus (IAV) and ferroptosis remains unclear. In this study, IAV hemagglutinin is shown to support viral replication by inducing ferroptosis via ferritinophagy. Hemagglutinin interacts with the autophagic receptors NCOA4 and TAX1BP1, leading to ferritinophagy. This interaction results in cellular lipid peroxidation and inhibits mitochondrial antiviral signaling protein (MAVS) aggregation, thereby promoting viral replication.

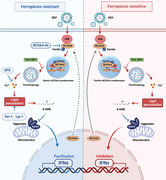

## 1 Introduction

Influenza A virus (IAV) is a segmented, single‐stranded, negative‐stranded RNA‐enveloped virus. It serves as a crucial zoonotic agent, causing acute respiratory infections that result in annual seasonal epidemics and occasional global pandemics, posing significant challenges to human and animal health.^[^ ^1^ ^]^ A major trigger for the influenza virus's pathogenicity is the excessive cellular inflammatory and immune response, which is closely related to cell death.^[^ ^2^, ^3^ ^]^ Previous studies have demonstrated that IAV induces several types of cell death, including apoptosis, necrosis, pyroptosis, and autophagy.^[^ ^4^, ^5^, ^6^ ^]^ Cell death plays a dual role in viral infections: while it protects the host from additional infections, it also promotes pathogenicity.^[^ ^7^, ^8^ ^]^ However, the cell death mechanisms caused by IAV and their encoded viral proteins have not been fully elucidated, and the significance of this process for viral infections remains unclear.

Ferroptosis represents a novel form of non‐apoptotic cell death characterized by iron‐dependent accumulation of lipid peroxides. The essence of ferroptosis lies in the peroxidation of polyunsaturated fatty acid‐containing phospholipids (PUFA‐PLs) catalyzed by the Fenton reaction into lipid peroxides and further lipid reactive oxygen species (ROS), which damage cell membranes and leads to cell death.^[^ ^9^ ^]^ The major drivers of ferroptosis include glutathione (GSH) depletion, lipid peroxides accumulation, and increased free iron levels.^[^ ^10^, ^11^ ^]^ Ferritin plays a crucial role in cellular iron homeostasis by storing excess free iron to protect cells from oxidative damage and releasing free iron upon rapid degradation in lysosomes.^[^ ^12^ ^]^ Nuclear receptor coactivator 4 (NCOA4) acts as a cargo receptor for ferritin autophagic degradation, thereby regulating intracellular iron homeostasis through ferritinophagy.^[^ ^13^ ^]^ Historically, ferroptosis has been linked to diseases such as tumors, organ damage, and neurodegenerative disorders.^[^ ^14^, ^15^ ^]^ However, recent reports indicate that viruses can also induce ferroptosis and enhance their pathogenicity through diverse pathways.^[^ ^16^, ^17^, ^18^ ^]^ Certain viruses manipulate ferroptosis to promote viral replication and spread, such as Coxsackievirus A6 (CV‐A6).^[^ ^19^ ^]^ While previous studies have suggested a potential role for ferroptosis in facilitating the replication of swine influenza viruses (SIV),^[^ ^20^ ^]^ the manipulation of ferroptosis by IAV remains a subject of debate, and the exact mechanism has yet to be elucidated.

The crucial role of RIG‐I‐like receptors (RLRs) in controlling RNA virus infections is well‐established.^[^ ^21^ ^]^ Mitochondrial antiviral signaling protein (MAVS) functions as a platform for RLR signaling, leading to the formation of prion‐like aggregates upon activation. These aggregates recruit downstream adaptor molecules that activate interferon regulatory factor 3 (IRF3) and induce type I interferon (IFN) production.^[^ ^22^ ^]^ MAVS activation is regulated by post‐translational modifications (PTMs), including ubiquitination, phosphorylation, and glycosylation, which have been reported to affect MAVS aggregation and protein stability.^[^ ^23^, ^24^, ^25^ ^]^ In addition, direct protein interactions, autophagy, and oxidative stress can also modulate MAVS signaling.^[^ ^26^, ^27^, ^28^ ^]^ IAV has evolved multiple strategies to evade MAVS‐mediated antiviral immunity. IAV PB1 protein ubiquitinates modified MAVS through RNF5 interactions and degrades MAVS by NBR1‐mediated selective autophagy.^[^ ^29^ ^]^ The IAV proteins NP and PB1‐F2 degrade MAVS by inducing mitophagy.^[^ ^30^, ^31^ ^]^ Moreover, the NS1 protein inhibits antiviral signaling by interfering MAVS interactions with TRAF3.^[^ ^32^ ^]^ However, it remains unclear whether other proteins of influenza A virus also exploit additional strategies to evade MAVS‐mediated antiviral immunity.

In this study, we demonstrated that IAV hemagglutinin (HA) induces cellular ferroptosis through ferritinophagy, leading to lipid peroxidation and impairing MAVS‐mediated type I IFN responses, thereby promoting viral replication. We discovered a mechanism of IAV‐induced ferroptosis, exploited by the virus to evade the host immune response.

## 2 Results

### 2.1 IAV Induces Cellular Ferroptosis In Vitro

**Figure 1:**
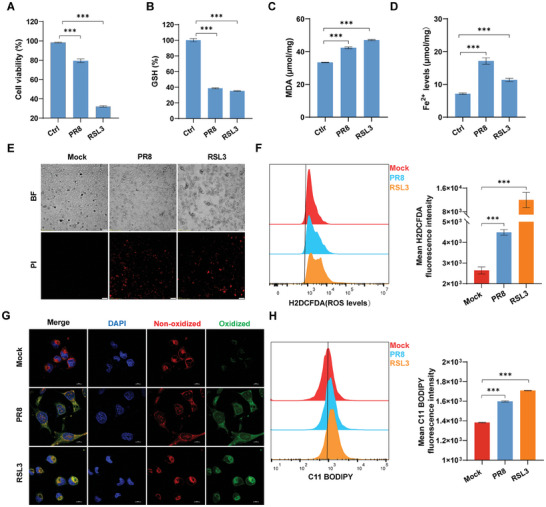
IAV induces cellular ferroptosis in vitro. A549 cells were treated with RSL3 (2 µm) or infected with PR8 H1N1 virus (MOI = 0.1) for 24 hpi. A) Cell viability was determined using a CCK‐8 kit. B) The intracellular GSH levels were determined by GSH assay kit. C) The cellular concentration of MDA was used to measure the level of lipid peroxidation by MDA assay kit. D) The ferrous iron concentration in cell lysates was determined by iron assay kits. E) The cell breakage was taken with PR8 H1N1 virus (MOI = 0.1) infection or RSL3 (2 µm) treatment. Cell morphology was observed in BF, and dead cells were labeled with PI. Scale bar = 100 µm. F) The H2DCFDA probe‐labeled intracellular ROS levels were detected by flow cytometry, and mean fluorescence intensity (MFI) was also analyzed. G and H) The C11 BODIPY probe stained intracellular lipid ROS, which were subsequently detected by confocal microscopy as well as flow cytometry, respectively, and the MFI of C11 BODIPY was also analyzed. Scale bar = 10 µm. Data were shown as means ± SEM (*n* = 3) from triplicate independent experiments, and significance was analyzed by two‐tailed Student's t‐test. (***p < 0.001).

IAV‐induced ferroptosis was rarely clear. Here, we evaluated the evidence of ferroptosis following IAV infection, as previously described.^[^ ^9^ ^]^ RSL3 was used as a positive control for ferroptosis and directly acted on GPX4 to induce ferroptosis.^[^ ^33^ ^]^ Consistent with RSL3 treatment, cell viability significantly decreased following PR8 H1N1 virus infection (**Figure** **1**A). Similarly to RSL3, PR8 H1N1 virus infection significantly reduced intracellular GSH levels (Figure 1B), as well as increased intracellular Malondialdehyde (MDA) (Figure 1C) and Fe^2+^ (Figure 1D) levels. To investigate the effects of IAV on cell death and morphology, cells with compromised membranes were stained with PI and visualized under fluorescence microscopy. In line with RSL3 treatment, PR8 H1N1 virus infection induced greater cell death (Figure 1E). Lipid peroxidation is caused by iron‐dependent excess ROS.^[^ ^35^ ^]^ We determined the intracellular ROS levels labeled with the H2DCFDA probe by flow cytometry. The results showed that PR8 H1N1 virus infection significantly increased ROS levels in A549 cells (Figure 1F). C11 BODIPY was used to determine lipid ROS levels. Flow cytometry and confocal microscopy results revealed that PR8 H1N1 virus infection significantly enhanced lipid ROS levels in A549 cells, reflecting evidence of lipid peroxidation (Figure 1G,H). This was also confirmed in NPTr cells. Consistent with RSL3 treatment, HuB H1N1 virus infection resulted in decreased newborn pig trachea (NPTr) cell viability (Figure S1A, Supporting Information), as well as reduced intracellular MDA and Fe^2+^ levels (Figure S1B,C, Supporting Information). The similarly further demonstrated that A549 cells are sensitive to IAV after infection using HM H5N1 virus (Figure S1D,E, Supporting Information). Taken together, these results demonstrated that IAV sensitizes cells to ferroptosis in vitro, particularly in IAV‐infected organ cells.

### 2.2 Inhibition of Ferroptosis Restricts IAV Replication

**Figure 2:**
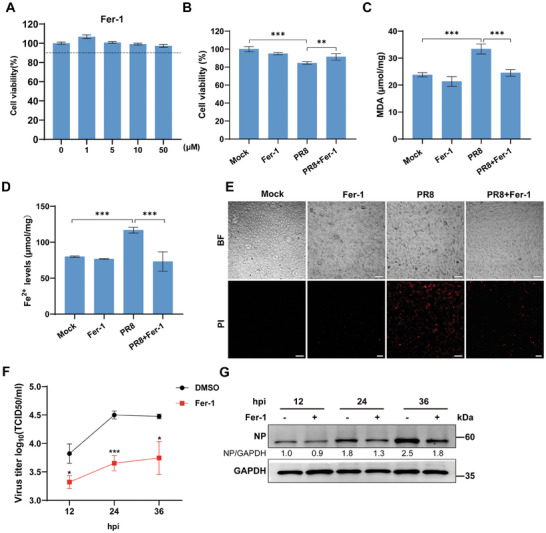
Inhibition of ferroptosis restricts IAV replication. A) The cell viability of A549 cells was measured by CCK‐8 after treatment with different concentrations of Fer‐1 for 24 h. B–E) A549 cells were pretreated with Fer‐1 (5 µm) or vehicle (DMSO) for 2 hpi, followed by infected A549 cells with PR8 H1N1 virus (MOI = 0.1) in Fer‐1 (5 µm) or vehicle (DMSO) treatment. B) Cell viability was determined using a CCK‐8 kit. C) The cellular concentration of MDA was used to measure the level of lipid peroxidation by MDA assay kit. D) The ferrous iron concentration in cell lysates was determined by iron assay kits. E)The cell breakage was visualized by fluorescence microscopy, the cell morphology was shown in BF, and the dead cells were stained by PI. Scale bar = 100 µm. F–G) A549 cells were pretreated with Fer‐1 (5 µm) or vehicle (DMSO) for 2 hpi, followed by infected A549 cells with PR8 H1N1 virus (MOI = 0.1) in Fer‐1 (5 µm) or vehicle (DMSO) treatment, and cell supernatants and lysates were harvested at 12, 24, and 36 hpi, respectively. F) Viral titers in MDCK cell supernatants were determined by TCID_50_. G) The viral protein NP expression detected by western blotting. Data were shown as means ± SEM (*n* = 3) from triplicate independent experiments, and significance was analyzed by two‐tailed Student's t‐test. (*p < 0.05; **p < 0.01; ***p < 0.001).

To further investigate whether inhibition of ferroptosis suppresses IAV‐induced lipid peroxidation and cell death, we used a lipid peroxidation inhibitor, Fer‐1,^[^ ^9^ ^]^ to attempt to rescue PR8 H1N1 virus‐infected A549 cells. With no significant effect of Fer‐1 on cell viability (**Figure** **2**A), the results showed that Fer‐1 treatment effectively enhanced cell viability (Figure 2B) and decreased MDA (Figure 2C) and Fe^2+^ (Figure 2D) levels after PR8 H1N1 virus infection. In the cell damage assay, Fer‐1 treatment likewise significantly reduced PI fluorescence intensity and cell damage after PR8 H1N1 virus infection (Figure 2E). After HuB H1N1 virus infection of NPTr cells, Fer‐1 treatment also improved cell viability (Figure S2A, Supporting Information) and decreased intracellular MDA and Fe^2+^ levels (Figure S2B,C, Supporting Information). In summary, the inhibition of ferroptosis significantly ameliorated IAV‐induced cell death, and consistent results were obtained in HM H5N1 virus infection (Figure S2D,E, Supporting Information). To clarify the correlation between IAV‐induced ferroptosis and the viral replication, we demonstrated that Fer‐1 treatment significantly reduced viral titer levels (Figure 2F) and viral NP levels (Figure 2G) at 12, 24, and 36 hours post‐infection (hpi) after PR8 H1N1 virus infection. In addition, Lip‐1, another lipid peroxidation inhibitor, was used to further validate the effect of ferroptosis on IAV replication (Figure S3A,B, Supporting Information). Consistent with Fer‐1 treatment, Lip‐1‐induced inhibition of ferroptosis effectively suppressed viral titers and viral NP levels during PR8 H1N1 virus infection. Overall, our data demonstrated that viral replication was suppressed by inhibiting IAV‐induced ferroptosis.

### 2.3 Ferritinophagy‐Induced Ferroptosis Benefits IAV Replication

**Figure 3:**
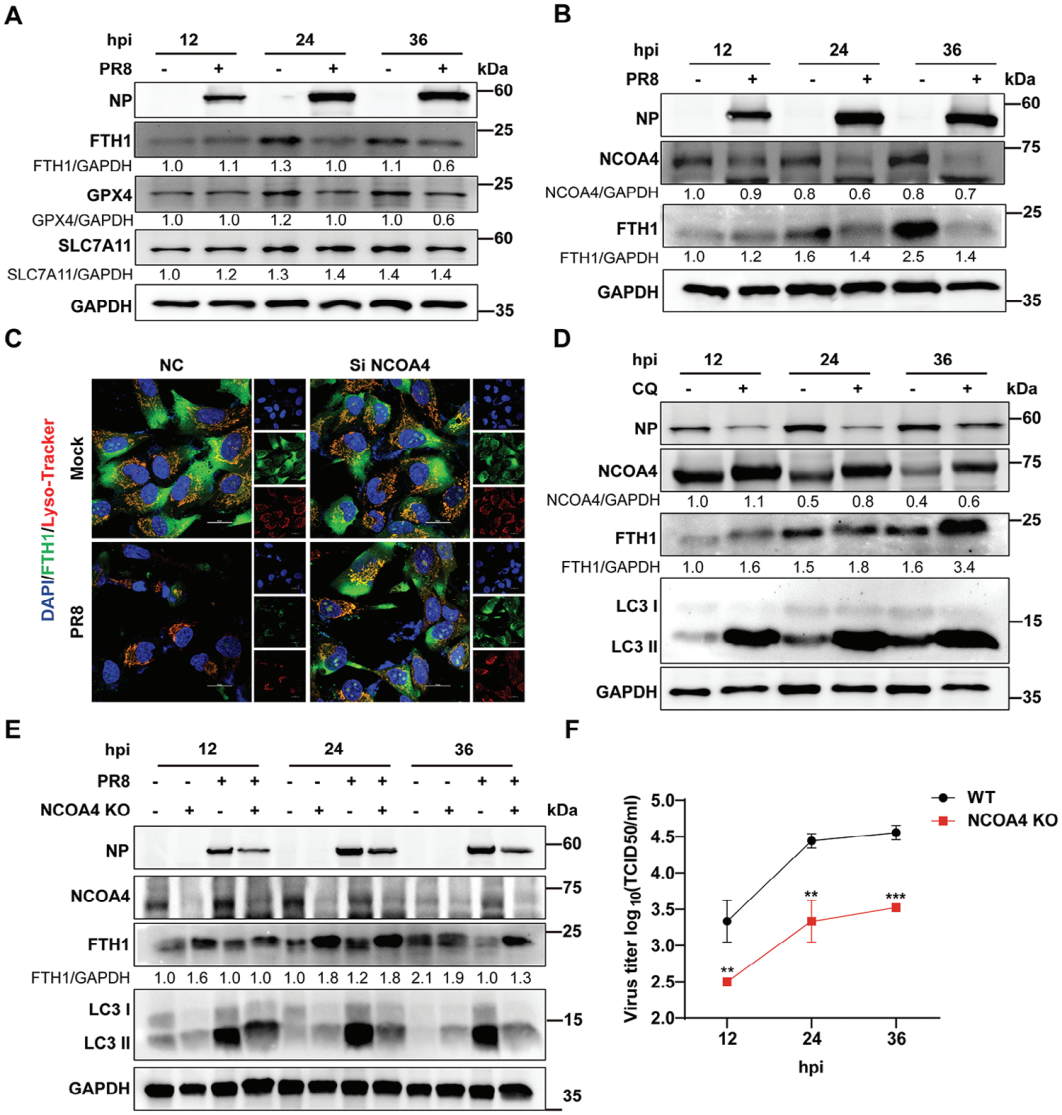
Ferritinophagy‐induced ferroptosis benefits IAV replication. A,B) The A549 cells were infected with PR8 H1N1 virus (MOI = 0.1) and cell lysates were harvested at 12, 24, and 36 hpi. A) The protein expression of ferroptosis genes was detected by western blotting with PR8 H1N1 infection of A549 cells. B) The protein expression of ferritinophagy genes was determined by western blotting with PR8 H1N1 virus infection of A549 cells. C) Confocal analysis of FTH1 expression levels in knockdown NCOA4 cells. Transfected with NC or Si NCOA4 in A549 cells, and infected with or without PR8 H1N1 virus (MOI = 0.1) 24 hpi following transfection, FTH1 (Green), lysosomes (Red), and cell nucleus (Blue) were stained. Scale bar = 20 µm. D) The protein expression of ferritinophagy genes was analyzed by western blotting for PR8 H1N1 virus infection treated with CQ. A549 cells infected with PR8 H1N1 virus (MOI = 0.1) at the treatment of CQ (100 µM) and cell lysates were harvested at 12, 24, and 36 hpi for western blotting analysis. E,F) PR8 H1N1 virus (MOI = 0.1) infected A549WT cells or NCOA4 KO cells, and cell supernatants as well as lysates were harvested. E) The protein expression of ferritinophagy genes and the viral nucleoprotein with PR8 H1N1 virus infection was analyzed by western blotting in NCOA4 KO cells. F) Viral titers in MDCK cell supernatants were determined by TCID_50_. Data were shown as mean ± SEM (*n* = 3) from triplicate independent experiments, and significance was analyzed by two‐tailed Student's *t*‐test. (**p < 0.01; ***p < 0.001).

In recent years, novel ferroptosis‐inducing pathways have been continuously identified, and the more classical pathways currently include: blockage of system Xc of cystine/glutamate reverse transporter proteins and inhibition of GSH synthesis, enhancement of lipid peroxidation and accumulation of lipid peroxidation products, accumulation of intracellular iron.^[^ ^36^, ^37^ ^]^ In particular, solute carrier family 7 member 11 (SLC7A11) is a key subunit of the systemic Xc reverse transporter protein complex, glutathione peroxidase 4 (GPX4) is a selenoprotein specialized in resisting lipid peroxidation by using GSH, and ferritin is a complex of proteins that are the main intracellular stores of iron, including ferritin heavy polypeptide 1 (FTH1) and ferritin light polypeptide 1 (FTL1).^[^ ^11^, ^38^ ^]^ To investigate the pathways involved in IAV‐induced ferroptosis, we first infected A549 cells with PR8 H1N1 virus and examined the protein expression levels of three key pathway genes at 12, 24, and 36 hpi, respectively. Western blotting showed a significant decrease in FTH1 and GPX4 protein expression levels after 24 hpi of PR8 H1N1 virus infection vs controls, which was consistent with ferroptosis characterization. No significant difference in SLC7A11 protein expression levels was observed (**Figure** **3**A). It was suggested that IAV may induce ferroptosis independently of the Xc system by affecting GSH utilization. Notably, the expression of FTH1, GPX4 as well as SLC7A11 was up‐regulated in the control group after 24 hpi, which was interpreted as related to the level of starvation^[^ ^39^ ^]^ and oxidative stress^[^ ^40^ ^]^ caused by the cells after serum‐free treatment. A similar result was demonstrated in HM H5N1 virus‐infected A549 cells (Figure S4A, Supporting Information). In NPTr cells, the expression of FTH1 and GPX4 was also significantly decreased at 24 and 36 hpi after HuB H1N1 virus infection. However, SLC7A11 showed no significant difference, consistent with the results in A549 cells (Figure S4B, Supporting Information). NCOA4 was reported to facilitate ferroptosis by participating in ferritin turnover through ferritinophogy.^[^ ^38^ ^]^ Thus, we verified whether PR8 H1N1 virus induces ferritinophogy. The results showed that NCOA4 protein expression was significantly down‐regulated in PR8 H1N1 virus‐infected cells consistent with FTH1 (Figure 3B). To validate whether PR8 H1N1 virus‐induced ferritinophogy is mediated by NOCA4, we silenced NCOA4 (Figure S4E, Supporting Information) and labeled lysosomes by Lyso‐Tracker to explore the degradation of FTH1 in lysosomes. In NC cells, FTH1 fluorescence was significantly attenuated after PR8 H1N1 virus infection, and silencing NCOA4 significantly inhibited the attenuation of FTH1 fluorescence (Figure 3C). It was also consistent with the results of the western blotting analysis (Figure S4F, Supporting Information). It indicated that NCOA4 mediated PR8 H1N1 virus‐induces ferritinophogy. Additionally, we also used an autophagy inhibitor, chloroquine (CQ), to validate PR8 H1N1 virus‐induced ferritinophogy under autophagy inhibition. As expected, CQ significantly inhibited FTH1 degradation caused by PR8 H1N1 virus infection, which significantly accumulated at 36 hpi (Figure 3D). In NPTr cells, we also investigated HuB H1N1 virus‐induced ferritinophagy. Consistent with the results in A549 cells, the protein expression of NCOA4 and FTH1 was significantly decreased at 12, 24, and 36 hpi after HuB H1N1 virus infection (Figure S4C, Supporting Information). In CQ‐treated NPTr cells, the degradation of NCOA4 and FTH1 was significantly inhibited after HuB H1N1 virus infection compared to the control (Figure S4D, Supporting Information). In addition, an NCOA4 knockout (KO) cell line was constructed in A549 cells (Figure S4G, Supporting Information). Protein expression levels were determined at 12, 24, and 36 hpi in NCOA4 KO or wild‐type (WT) cells infected with PR8 H1N1 virus. The results showed that FTH1 turnover was inhibited and protein expression accumulated after NCOA4 KO. NCOA4 KO significantly inhibited FTH1 degradation after PR8 H1N1 virus infection. Interestingly, viral NP protein levels were also significantly reduced by NCOA4 KO (Figure 3E). We further examined the viral titers in the supernatants of NCOA4 KO and WT cells after viral infection, which were consistent with the western blotting results that NCOA4 KO significantly inhibited PR8 H1N1 viral replication (Figure 3F). NCOA4‐9a was newly identified as an inhibitor of ferroptosis, which inhibited ferritinophogy by competitively inhibiting the interaction of NCOA4 with FTH1.^[^ ^41^ ^]^ With NCOA4‐9a treatment, NP protein expression of PR8 H1N1 virus in A549 cells was significantly inhibited (Figure S5A, Supporting Information) and the viral titer in the supernatant was also significantly decreased (Figure S5B, Supporting Information). In previous results, we observed a significant reduction in GPX4 protein expression with PR8 H1N1 virus infection. Hence, we verified the effect of silencing GPX4 against PR8 H1N1 virus replication (Figure S5C, Supporting Information). The results demonstrated that the silencing of GPX4 in A549 cells had no significant effect on NP protein expression (Figure S5D, Supporting Information) vs viral titer (Figure S5E, Supporting Information) of PR8 H1N1 virus. Overall, our results demonstrated that IAV‐induced NCOA4‐mediated ferritinophogy resulted in sensitivity to ferroptosis. Moreover, the suppression of NCOA4‐mediated ferritinophogy significantly inhibited the replication of IAV.

### 2.4 Hemagglutinin Induces Ferritinophagy by Interacting with TAX1BP1 and NCOA4

**Figure 4:**
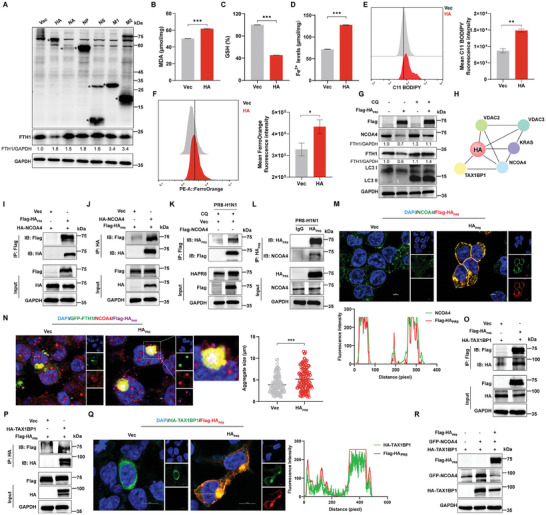
Hemagglutinin induces ferritinophagy by interacting with TAX1BP1 and NCOA4. A) The degradation of ferritin by PR8 H1N1 viral proteins. HEK293T cells were transfected with nine plasmids encoding PR8 H1N1 viral proteins, and cell lysates were harvested at 24 h for western blotting to detect the protein expression of FTH1. *Target bands. B–F) HEK293T cells were transfected with PR8 HA, and cells or cell lysates were harvested at 24 h post‐transfection. B) Intracellular MDA levels were determined by MDA kit. C) Cellular GSH levels were determined by GSH assay kit. D) Intracellular ferrous iron concentration was determined by iron assay kit. E) Intracellular lipid ROS levels were determined with C11 BODIPY probe staining by flow cytometry. F) Intracellular free ferrous iron was determined with FerroOrange probe staining by flow cytometry. The MFI of C11 BODIPY and FerroOrange were analyzed. G) The protein expression of ferritinophagy genes following transfection with PR8 HA in case of autophagy inhibition HEK293T cells were transfected with PR8 HA for 6 h and then treated with or without CQ (100 µm) for 24 h, the cell lysates were harvested by western blotting analysis. H) An interaction network analysis of PR8 HA. I,J) The exogenous interactions of PR8 HA with NCOA4. HEK293T cells were transfected with Flag‐HA_PR8_ and HA‐NCOA4, and cell lysates were harvested at 24 h for Co‐IP and western blotting analysis. K,L) The endogenous interactions of PR8 HA with NCOA4. K) The Flag‐NCOA4 was transfected in HEK293T cells, treated with CQ (100 µm) for 24 h at 6 h post‐transfection, cell lysates were used for Flag‐NCOA4 pull‐down and western blotting analysis. L) PR8 H1N1 virus (MOI = 0.1) was infected with HEK293T cells, and cell lysates used in PR8 HA pull‐down and western blotting analysis. M) Confocal analysis of the co‐localization of PR8 HA with NCOA4. HEK293T cells were transfected with vector or Flag‐HA_PR8_ (Red), then indicated with the relevant antibodies for Flag‐HA_PR8_ (Red) and NCOA4 (Green), the cell nucleus was stained with DAPI (Blue). The fluorescence intensity profiles of Flag‐HA_PR8_ and NCOA4 were measured along the lines drawn by Image J. Scale bar = 5 µm. N) The effects of PR8 HA on Ferritin‐NCOA4 condensates. HEK293T cells were transfected with a vector or Flag‐HA_PR8_ and GFP‐FTH1 (Green). NCOA4 (Red) and Flag‐HA_PR8_ (Violet) were detected using specific antibodies, and the cell nuclei were stained with DAPI. Subsequently, 100 condensates with diameters larger than 1 µm were randomly statistically analyzed. Scale bar = 2 µm. O,P) The exogenous interaction of the PR8 HA with TAX1BP1. HEK293T cells were transfected with Flag‐HA_PR8_ and HA‐TAX1BP1, followed by harvesting of cell lysates for Co‐IP and western blotting analysis. Q) Confocal analysis of co‐localization for PR8 HA with TAX1BP1. HEK293T cells were transfected with vector or Flag‐HA_PR8_ and HA‐TAX1BP1, the relevant antibodies indicated Flag‐HA_PR8_ (Red), HA‐TAX1BP1 (Green), and the cell nucleus was stained with DAPI. The fluorescence intensity profiles of Flag‐HA_PR8_ and HA‐TAX1BP1 were measured along the lines drawn by Image J. Scale bar = 10 µm. R) The effect of PR8 HA with regard to the protein expression of TAX1BP1‐NCOA4. HEK293T cells were transfected with vector or HA‐NCOA4 and Flag‐TAX1BP1, and cell lysates were analyzed by western blotting with or without Flag‐HA_PR8_ present. Data were shown as mean ± SEM (*n* = 3) from triplicate independent experiments, and significance was analyzed by two‐tailed Student's *t*‐test. (*p < 0.05; **p < 0.01; ***p < 0.001).

To investigate the exact molecular mechanism of IAV‐induced ferritinophogy, we initially screened the viral proteins for their role in IAV‐induced ferritinophagy. The major viral proteins (HA, NA, NP, NS, M1, and M2, other viral proteins data not shown) of PR8 H1N1 virus were transfected in HEK293T cells. It was interesting to recognize that the degradation of FTH1 was most pronounced after transfection of PR8 HA (**Figure** **4**A). Next, we verified whether PR8 HA induced cellular ferroptosis as well. The PR8 HA was transfected into HEK293T cells. The cellular levels of MDA, GSH, and Fe^2+^ were determined separately. It was shown that PR8 HA transfection resulted in increased levels of MDA, Fe^2+^, lipid ROS, and unstable ferrous iron, and decreased levels of GSH in the cells (Figure 4B–F). All these results indicated that IAV hemagglutinin induced ferroptosis in the cells. The PR8 HA was previously observed to induce degradation of FTH1, and thus we verified PR8 HA‐induced ferritinophogy with CQ treatment. As expected, the NCOA4 and FTH1 protein expression was downregulated with PR8 HA transfection, which led to the onset of cellular ferritinophogy. And FTH1 degradation was significantly inhibited by CQ treatment and NCOA4 was partially restored (Figure 4G). We also explored the effect of PR8 HA on cellular ferritinophogy in KO (Figure S4H, Supporting Information) or knockdown of NCOA4 cells. Similar to CQ treatment, FTH1 degradation was significantly inhibited by NCOA4 KO or knockdown (Figure S6A,B, Supporting Information). It was indicated that IAV‐induced ferritinophagy is induced by hemagglutinin. However, the specific mechanism of how hemagglutinin induces cellular ferritinophogy is not clear. We thus enriched the reciprocal host proteins of PR8 HA by Co‐IP and determined the interacting proteins of the ferroptosis pathway by LC‐MS/MS analysis. The mass spectrometry identified 227 host proteins that specifically interact with PR8 HA and enriched them in the ferroptosis pathway (Figure S6C,D, Supporting Information), and we preliminarily screened for host proteins that interact with PR8 HA: NCOA4, tax1‐binding protein 1 (TAX1BP1), VDAC2/3, etc (Figure 4H). Then we first verified the interactions between PR8 HA and NCOA4 by Co‐IP. The exogenous PR8 HA and NCOA4 plasmids were transfected in HEK293T cells, respectively. The results showed that Flag‐HA_PR8_ interacted with HA‐NCOA4 (Figure 4I,J). Similarly, the virus‐derived HA protein interacted with NCOA4 in PR8 H1N1 virus‐infected cells (Figure 4K,L). The co‐localization of PR8 HA with NCOA4 was also investigated. The results showed that Flag‐HA_PR8_ co‐localized almost fully with NCOA4 at the cell membrane with the cytoplasm (Figure 4M). This led to new doubts that PR8 HA interacts with NOCA4 for ferritinophagy the meaning of which remains unclear. While we noticed that PR8 HA migrated the subcellular localization of NCOA4 in the above co‐localization data. NCOA4 was mainly localized to the nucleus and cytoplasm, and nuclear localization signals of NCOA4 were reduced toward the cytoplasm and cytosol by transfection of PR8 HA in HEK293T cells or infection with PR8 H1N1 virus in A549 cells (Figure S6E, Supporting Information). It's demonstrated that NCOA4 in the nucleus is recruited by the PR8 HA thereby facilitating ferritinophagy. Ferritin has been described as degraded by autophagy through the formation of ferritin‐NCOA4 condensates in an NCOA4‐dependent manner.^[^ ^42^ ^]^ We analyzed the co‐localization of PR8 HA, FTH1, and NCOA4 after transfection with vector or Flag‐HA_PR8_ in HEK293T cells. With transfection of Flag‐HA_PR8_, consistent with the above, nuclear localization of NCOA4 was reduced and PR8 HA, FTH1 and NCOA4 co‐localized intracellularly. Importantly, a large fluorescent spot was formed by FTH1 with NCOA4, which suggested the formation of a ferritin‐NCOA4 condensate. Subsequently, we randomly counted 100 fields of view and defined fluorescent spots with a diameter larger than 1 µm as ferritin‐NCOA4 condensates.^[^ ^42^ ^]^ The number of ferritin‐NCOA4 condensates was significantly increased in cells transfected with Flag‐HA_PR8_ (Figure 4N). The previous findings demonstrated that TAX1BP1 mediated the degradation of ferritin‐NCOA4 condensate into the lysosomes.^[^ ^42^, ^43^ ^]^ Moreover, the LC–MS/MS identified PR8 HA interacting with TAX1BP1 (Figure 4H). Next, the interaction of HA‐TAX1BP1 with Flag‐HA_PR8_ was demonstrated by Co‐IP (Figure 4O,P), and the co‐localization of HA‐TAX1BP1 with Flag‐HA_PR8_ in HEK293T cells was also demonstrated. In transfected Flag‐HA_PR8_ cells, the fluorescent spots of HA‐TAX1BP1 also showed aggregation (Figure 4Q). In addition, AlphaFold3 was used to clearly visualize the interactions among the structures of PR8 HA, NCOA4, and TAX1BP1. Further analysis of the interaction residues of the predicted molecular model complexes revealed interactions between the C‐terminus of PR8 HA, the N‐terminus of NCOA4, and the N‐terminus of TAX1BP1 involving intermolecular forces (Figure S6F, Supporting Information). Compared to the vector group, HA‐TAX1BP1 enhanced the expression of GFP‐NCOA4, while the addition of Flag‐HA_PR8_ resulted in a significant down‐regulation of GFP‐NCOA4 expression in the presence of HA‐TAX1BP1 (Figure 4R). It's indicated that PR8 HA promoted TAX1BP1‐mediated ferritinophogy. In conclusion, our results suggested that IAV hemagglutinin promotes TAX1BP1‐mediated autophagic degradation of ferritin‐NCOA4 condensate by interacting with NCOA4 and TAX1BP1.

### 2.5 Lipid Peroxidation Caused by IAV‐Induced Ferritinophagy Inhibits IFN‐β Levels

**Figure 5:**
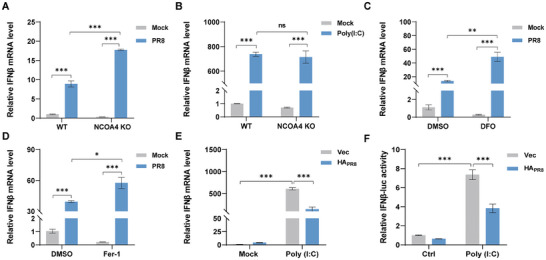
Lipid peroxidation caused by IAV‐induced ferritinophagy inhibits IFN‐β levels. A) qRT‐PCR analysis of IFNβ mRNA levels by PR8 H1N1 virus‐stimulated in NCOA4 KO cell lines. A549 WT or NCOA4 KO cells were infected with or without PR8 H1N1 virus (MOI = 0.1) for 24 hpi. qRT‐PCR analysis of IFNβ mRNA levels was performed. B) qRT‐PCR analysis of IFNβ mRNA levels in NCOA4 KO cells with Poly (I:C)‐stimulated. A549 WT or NCOA4 KO cells were stimulated with Poly (I:C) (200 ng) for 12 h. qRT‐PCR analysis of IFNβ mRNA levels was performed. C,D) IFNβ mRNA levels by PR8 H1N1 virus (MOI = 0.1) stimulated A549 cells with or without lipid peroxidation inhibitor treatment. C) A549 cells with or without DFO (100 µm) treatment, and the IFNβ mRNA levels were analyzed by qRT‐PCR. D) A549 cells with or without Fer‐1 (5 µm) treatment. the IFNβ mRNA levels were analyzed by qRT‐PCR. E,F) HEK293T cells were transfected with PR8 HA, followed by Poly (I:C) (200 ng) stimulation for 12 h, and IFNβ levels were analyzed by qRT‐PCR assay and luciferase assay, respectively. Data were shown as means ± SEM (*n* = 3) from triplicate independent experiments, and significance was analyzed by two‐tailed Student's t‐test. (*p < 0.05; **p < 0.01; ***p < 0.001; ns, no significant).

Previous findings have demonstrated that inhibition of ferritinophagy‐induced ferroptosis suppresses IAV replication, but the specific pathways by which ferritinophogy affects IAV replication remain unclear. We first explored the type I IFN response in NCOA4 KO cells with PR8 H1N1 virus‐infection. Compared to WT cells, NCOA4 KO significantly boosted mRNA levels of IFNβ (**Figure** **5**A). In contrast, there was no significant difference in the mRNA levels of IFNβ between WT or NCOA4 KO cells following Poly (I:C) stimulation (Figure 5B). This suggested that the increase of IFNβ mRNA levels by NCOA4 KO was due to the suppression of ferritinophogy induced by IAV. It is known that ferritinophogy occurs with the degradation of ferritin, which releases a large amount of Fe^2+^, they participate in the Fenton reaction, generating oxygen radicals represented by hydroxyl radicals, which peroxidize the membrane lipids and cause cell death.^[^ ^34^ ^]^ Hence, we investigated whether ferritinophogy influences type I interferon response via intracellular Fe^2+^ levels and lipid peroxidation levels to the detriment of IAV replication. The A549 cells were treated with the iron chelator DFO and the lipid peroxidation inhibitor Fer‐1, respectively. Both DFO and Fer‐1 treatment significantly enhanced the mRNA IFNβ levels with PR8 H1N1 virus infection (Figure 5C,D). In addition, we combined flow cytometry and confocal analysis that NCOA4 KO significantly reduced Fe^2+^ levels (Figure S7A–D, Supporting Information) and lipid ROS levels (Figure S7E,F, Supporting Information) in PR8 H1N1 virus‐infected or PR8 HA‐transfected cells. It also demonstrated that the inhibition of ferritinophogy ultimately suppressed cellular lipid peroxidation levels. Taken together, we found that inhibition of cellular lipid peroxidation levels due to ferritinophogy suppressed IFNβ mRNA levels in PR8 H1N1 virus‐infected cells. Furthermore, we also verified whether PR8 HA‐induced lipid peroxidation affected type I IFN responses. The results showed that PR8 HA likewise significantly inhibited Poly (I:C)‐stimulated cellular IFNβ promoter activity (Figure 5E) and mRNA levels (Figure 5F). The reason for this may be due to the fact that hemagglutinin inhibits IFNβ levels via ferritinophagy‐induced cellular lipid peroxidation.

### 2.6 Lipid Peroxidation Resulting from Hemagglutinin Impairs MAVS‐Mediated Antiviral Immunity

**Figure 6:**
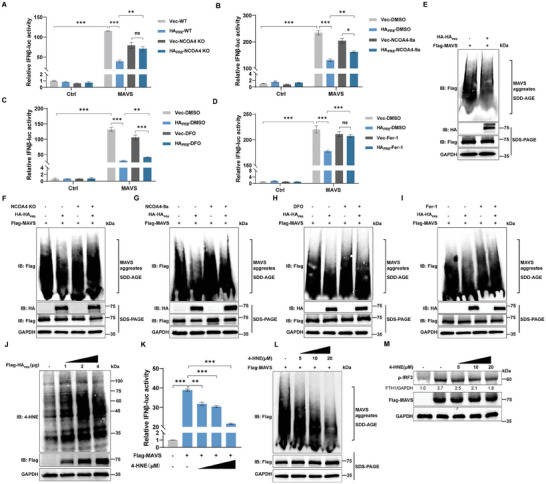
Lipid peroxidation resulting from IAV hemagglutinin impairs MAVS‐mediated antiviral immunity. A) The IFNβ promoter activity induced by MAVS in HEK293T WT cells or NCOA4 KO cells, with or without transfection of PR8 HA. HEK293 WT or KO cells were transfected with vector or PR8 HA and MAVS, followed by IFNβ promoter activity determined by luciferase assay. B–D) The effects of PR8 HA on MAVS‐induced IFNβ promoter activity with and without lipid peroxidation inhibition. HEK293T cells were transfected with either vector or PR8 HA and MAVS. After 6 h post‐transfection, the cells were treated with NCOA4‐9a (2.5 µm), DFO (100 µm), or Fer‐1 (5 µm) for 24 h. The IFNβ promoter activity was detected by luciferase assay. E) Western blotting analysis was performed to investigate the impact of PR8 HA on MAVS aggregates. HEK293T cells were co‐transfected with vector or HA‐HA_PR8_ and Flag‐MAVS in HEK293T cells, and cell lysates were harvested at 24 h for SDS‐PAGE and SDD‐AGE, followed by western blotting analysis. F) Western blotting analysis of the effect of PR8 HA for MAVS aggregates in NCOA4 KO cells. In HEK293T WT or NCOA4 KO cells, vector or HA‐HA_PR8_ and Flag‐MAVS were co‐transfected separately, and cell lysates were harvested at 24 h post‐transfection for SDD‐AGE vs SDS‐PAGE, followed by western blotting analysis. G‐I) The effects of PR8 HA on MAVS aggregates with and without lipid peroxidation inhibition. HEK293T cells were transfected with either vector or PR8 HA and MAVS. After 6 h of transfection, the cells were treated with NCOA4‐9a (2.5 µm), DFO (100 µm) or Fer‐1 (5 µm) for 24 h. Cell lysates were harvested at 24 h post‐transfection for SDD‐AGE vs SDS‐PAGE, followed by western blotting analysis. J) 4‐HNE levels were analyzed by western blotting in HEK293T cells transfected with 1, 2, and 4 µg of PR8 HA. K‐M) HEK293T cells were transfected with Flag‐MAVS, followed by treatment with 0, 5, 10 and 20 µm 4‐HNE for 12 h. Cell lysates were harvested for analysis. K) The IFNβ promoter activity induced by 4‐HNE on MAVS was determined by luciferase assay. L) The effects of 4‐HNE on MAVS aggregates were analyzed by western blotting. M) The ρ‐IRF3 level induced by 4‐HNE on MAVS was analyzed by western blotting. Data were shown as mean ± SEM (*n* = 3) from triplicate independent experiments, and significance was analyzed by two‐tailed Student's t‐test. (**p < 0.01; ***p < 0.001; ns, no significant).

To further investigate whether the hemagglutinin of IAV affects the specific mechanism of type I IFN response through lipid peroxidation due to ferritinophagy. MAVS, a key signaling protein against RNA viruses, was used to investigate the effect of IAV hemagglutinin on type I IFN responses.^[^ ^44^ ^]^ Consistent with previous results, PR8 HA also inhibited MAVS‐mediated IFNβ promoter activity, but the functional blocking of ferritinophagy ameliorated this inhibition with NCOA4 KO or NCOA4‐9a treatment (**Figure** **6**A,B). Similarly, treatment with DFO or Fer‐1 significantly ameliorated the inhibition of MAVS‐mediated IFNβ promoter activity by PR8 HA (Figure 6C,D). PR8 HA impaired the MAVS‐mediated type I IFN response through lipid peroxidation caused by ferritinophogy, which corroborates our previous findings. MAVS aggregation is critical for the activation of antiviral innate immunity downstream by RIG‐I‐like receptors (RLRs).^[^ ^44^ ^]^ To delve deeper into the fact that PR8 HA impairs MAVS‐mediated type I IFN response, the effect of PR8 HA on MAVS aggregation was examined. As expected, PR8 HA suppressed the aggregation of MAVS (Figure 6E). Consistent with the results of previous luciferase assays, NCOA4 KO or NCOA4‐9a treatment also ameliorated the attenuation of MAVS aggregation by PR8 HA (Figure 6F,G). The treatment of DFO or Fer‐1 equally ameliorated the impairment of MAVS aggregation by PR8 HA (Figure 6H,I). Notably, compared to treatment with DFO, the enhancement of MAVS‐mediated type I IFN responses was more effective with NCOA4 KO, NCOA4‐9a or Fer‐1 treatment. Overall, these results demonstrated that PR8 HA‐induced ferritinophagy leading to lipid peroxidation is an important causal factor in impairing the antiviral immunity of MAVS. However, the mechanism by which lipid peroxidation impairs MAVS‐mediated antiviral immunity remains elusive. The cellular process of lipid peroxidation converts membrane lipids into a series of lipid peroxidation products, mostly including 4‐HNE and MDA.^[^ ^9^, ^45^ ^]^ A progressive increase of the cellular 4‐HNE level was observed with an increasing dose of Flag‐HA_PR8_ transfected in HEK293T cells (Figure 6J). Next, 4‐HNE was used to evaluate the role of lipid peroxidation on MAVS‐mediated antiviral immunity. The MAVS‐mediated IFNβ promoter activity was inhibited by 4‐HNE in a dose‐dependent manner (Figure 6K). 4‐HNE also impaired MAVS aggregation (Figure 6L) and inhibited MAVS‐mediated downstream IRF3 phosphorylation (Figure 6M). These data indicated that IAV hemagglutinin impaired MAVS aggregation and suppressed downstream type I IFN responses via cellular lipid peroxidation products, such as 4‐HNE.

### 2.7 IAV Hemagglutinin‐Induced Lung Ferroptosis Impairs MAVS in Vivo

**Figure 7:**
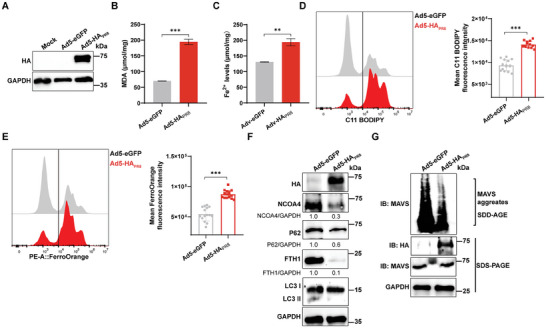
IAV hemagglutinin‐induced lung ferroptosis impairs MAVS in vivo. A) The expression of Ad5‐HA_PR8_ in A549 cells was analyzed by western blotting. The recombinant adenoviruses Ad5‐eGFP and Ad5‐HA_PR8_ infected A549 cells (MOI = 0.1) and cell lysates were harvested at 48 hpi. Mock, Ad5‐eGFP served as a negative control. B‐G) Mice were infected with Ad5‐eGFP and Ad5‐HA_PR8_, and ferroptosis was examined in mice lungs at 3 days (*n* = 5). B) Intracellular MDA levels were determined by MDA kit. C) Intracellular ferrous iron concentration was determined by iron assay kit. D) Intracellular lipid ROS levels were determined with C11 BODIPY probe staining by flow cytometry. E) Intracellular free ferrous iron was determined with FerroOrange probe staining by flow cytometry. The MFI of C11 BODIPY and FerroOrange were analyzed. F) Western blotting analysis of protein expression of ferritinophagy genes in mice lungs following Ad5‐HA_PR8_ infection. G) Western blot analysis of the effects of MAVS aggregates in mice lungs after Adv‐HA_PR8_ infection. Data were shown as mean ± SEM, and significance was analyzed by two‐tailed Student's *t*‐test. (**p < 0.01; ***p < 0.001).

To investigate IAV hemagglutinin‐induced ferroptosis in vivo, we used recombinant adenovirus to intranasally inoculate mice that express the PR8 HA protein in their lungs. We first validated the expression of the recombinant adenovirus Ad5‐HA_PR8_ in lung cells by infecting A549 cells with Ad5‐eGFP or Ad5‐HA_PR8_. The results showed that Ad5‐HA_PR8_ induced high expression of PR8 HA protein in A549 cells in vitro compared to Ad5‐eGFP group (**Figure** **7**A). In recombinant adenovirus‐infected mice, the Ad5‐HA_PR8_ group significantly increased MDA and Fe^2+^ levels in the lungs (Figure 7B,C). Lipid ROS and unstable ferrous ion levels in isolated mouse lung tissue cells were detected by flow cytometry. The Ad5‐HA_PR8_ group significantly increased lipid ROS and unstable ferrous iron levels in the lungs (Figure 7D,E). Consistent with the in vitro results, these findings demonstrated that IAV hemagglutinin induces ferroptosis in lung tissue in vivo. Among previous results, it was shown that IAV hemagglutinin induces cellular ferritinophagy, impairing MAVS‐mediated antiviral immunity in vitro. A consistent conclusion was also demonstrated by the results of the in vivo experiments. The protein expression of NCOA4 and FTH1 was significantly reduced in the lungs of the Ad5‐HA_PR8_ group compared to the Ad5‐eGFP group. Additionally, P62 and LC3 indicated the occurrence of autophagy (Figure 7F). In conclusion, these findings demonstrated that PR8 HA induced ferritinophagy in the lungs in vivo. Consistent with the in vitro results, PR8 HA also suppressed MAVS aggregation in the lung (Figure 7G). Collectively, these results showed that PR8 HA induced ferroptosis through ferritinophagy in vivo and inhibited MAVS aggregation.

## 3 Discussion

IAV, a respiratory pathogen infecting both humans and animals, poses significant morbidity and lethality worldwide. Cell death is regarded as a suicidal protective mechanism for host clearance of pathogens.^[^ ^46^ ^]^ IAV enhances its pathogenicity and facilitates spread or replication by regulating cell death pathways.^[^ ^47^ ^]^ Ferroptosis, a recently identified mode of cell death, has garnered considerable attention.^[^ ^9^ ^]^ A few have reported that IAV induces ferroptosis.^[^ ^20^, ^48^ ^]^ However, the mechanism underlying IAV regulation of ferroptosis remains unclear. In this study, we demonstrated the mechanism of molecular interactions by which IAV induces cellular ferroptosis through the ferritinophagy pathway. Importantly, inhibition of the cellular ferroptosis pathway not only suppressed virus‐induced cell death, but also suppressed viral replication. To investigate the replication strategy of IAV exploiting ferritinophagy, we examined the impact of IAV exploiting ferritinophagy on IFN signaling in terms of antiviral immunity mediated by RLRs. Our results suggested that IAV hemagglutinin facilitates viral replication by inducing cellular lipid peroxidation, impairing MAVS aggregation, and inhibiting type I IFN response.

The induction of cell death by various pathogens facilitates their reproduction and pathogenic mechanisms.^[^ ^7^ ^]^ Ferroptosis, identified as a novel form of iron‐dependent lipid peroxidation‐driven cell death,^[^ ^9^ ^]^ has been reported to be manipulated by pathogens through multiple pathways. Of these, iron regulation and lipid metabolism are crucial pathways for the occurrence of ferroptosis. Certain viruses have been reported to manipulate the transferrin receptor (TFRC) or ferritinophagy to regulate intracellular free iron, thereby promoting the Fenton reaction and initiating ferroptosis.^[^ ^18^, ^49^, ^50^ ^]^ IAV can also manipulate cellular iron metabolism. TFRC and transferrin (TF) are significantly up‐regulated in cells after viral infection, and intracellular iron is also elevated.^[^ ^20^ ^]^ Consistently, our findings both in vitro and in vivo indicated that IAV regulates intracellular ferritin turnover through ferritinophagy to initiate the occurrence of ferroptosis. Ferritin is transported for lysosomal degradation via two pathways (macroautophagy and the ESCRT‐dependent endosomal microautophagy pathway), both requiring NCOA4 as the ferritinophagy receptor. NCOA4 provides multivalent interactions as a driver of ferritin‐NCOA4 condensate formation, mediated by TAX1BP1 incorporation into autophagosomes and endosomes.^[^ ^42^, ^43^ ^]^ In this study, we found that IAV hemagglutinin acts as a bridging protein between NCOA4 and TAX1BP1, recruiting NCOA4, which facilitates the formation of ferritin‐NCOA4 condensates that link to TAX1BP1 targeting lysosomal degradation and releasing Fe^2+^, thereby inducing cellular ferroptosis. Notably, the lipidation of LC3II in IAV hemagglutinin‐induced ferritinophagy was not identical to classical autophagy characteristics, implying that hemagglutinin‐induced ferritinophagy may be related to nonclassical autophagy via the ESCRT pathway, which does not rely on LC3. Additionally, IAV regulates various stages of its viral life cycle through autophagy to facilitate its replication.^[^ ^6^ ^]^ This is further evidenced by the utilization of ferritinophagy by IAV hemagglutinin to promote viral replication. The peroxidation of PUFA‐PLs‐containing lipids and the formation of lipid hydroperoxides distinguish ferroptosis from other types of cell death,^[^ ^51^, ^52^ ^]^ highlighting the importance of lipid metabolism in IAV‐induced ferroptosis. ACSL4 have been shown to drive ferroptosis by activating and facilitating the incorporation of PUFAs into membrane lipids (for example, phospholipids), whereas CV‐6 utilizes ACSL4 to manipulate lipid metabolism to promote the formation of viral replication organelle.^[^ ^19^ ^]^ Considering IAV as an enveloped virus utilizing large amounts of lipids as vesicles and energy sources.^[^ ^53^ ^]^ Combined with metabolomics, it was shown that IAV remodeled the lipid metabolism of host cells after infection. This suggests that IAV may manipulate lipid metabolism to induce cellular ferroptosis in addition to regulating cellular iron abundance and inhibiting GPX4‐dependent antioxidant pathways.

Interferon is the gateway to host defense against viral infection during viral infection. Pathogen‐associated molecular patterns recognized by RLRs interact with MAVS and activate downstream signaling, triggering a type I IFN response.^[^ ^21^ ^]^ MAVS serves as a key carrier switch in most anti‐RNA virus immune signaling, with a variety of evidence suggesting that its immune homeostasis is linked to ferroptosis.^[^ ^54^ ^]^ ROS accumulation, mitochondrial homeostasis imbalance, and iron metabolism all induce cellular ferroptosis in different ways that also affect MAVS activation. On the one hand, ROS promote cellular ferroptosis by depleting GSH and promoting the onset of lipid peroxidation, and on the other hand, elevated ROS affect MAVS aggregation.^[^ ^27^ ^]^ The regulation of mitochondrial activity also affects cellular sensitivity to ferroptosis, such as the metalloendopeptidases OMA1 and DAP3 binding cell death enhancer 1 (DELE1), which mediate integrated mitochondrial stress, the biosynthesis of mitochondrial Fe–S clusters, the mitochondrial fusion protein MFN1, etc.^[^ ^55^, ^56^, ^57^ ^]^ Similarly, mitochondrial homeostasis affects the MAVS protein stabilization and aggregation, such as mitochondrial fission and fusion, mitophagy, etc.^[^ ^30^, ^58^ ^]^ In addition, there is a report showing that the iron metabolism‐related gene Hfe mediates autophagic degradation of MAVS by interacting with SQSTM1/P62.^[^ ^59^ ^]^ In the current study, we found that IAV hemagglutinin inhibits MAVS aggregation by ferritinophagy. To investigate which specific process of hemagglutinin‐induced ferritinophagy affected MAVS activation, we examined the effect of hemagglutinin on MAVS‐mediated IFNβ levels under inhibition of ferritinophagy, chelation of iron, and inhibition of lipid peroxidation. The results showed that the inhibition of MAVS by hemagglutinin was ameliorated by these treatments. Lipid peroxidation, which is the end‐phase response to ferritinophagy‐induced ferroptosis, was identified as a key causative agent for the inhibition of MAVS activation by hemagglutinin‐induced ferritinophagy. 4‐HNE, a well‐known lipid peroxide and important marker of ferroptosis, acts as a highly reactive lipid aldehyde, forming adducts with macromolecules, including DNA, lipids, and proteins, leading to their carbonylation and disruption of their structure and function. In this study, the lipid peroxidation product 4‐HNE was shown to inhibit MAVS aggregation and suppress its downstream IFN response, which may be directly responsible for the inhibition of MAVS activation by lipid peroxidation. It is corroborated in HSV‐1‐induced lipid peroxidation, where 4‐HNE inhibited STING activation by inducing carbonylation of the C88 site of STING.^[^ ^60^ ^]^ However, the carbonylation of MAVS induced by 4‐HNE has not been validated, with only evidence of MAVS carbonylation modification in the raw identification data from previous studies of global mapping of protein carbonylation in ferroptosis.^[^ ^61^ ^]^

IAV hemagglutinin, a glycoprotein on the membrane of the viral vesicle, has been described to trigger endoplasmic reticulum stress,^[^ ^62^ ^]^ a crucial regulator of ferroptosis and the primary site for initiating membrane lipid peroxidation.^[^ ^10^ ^]^ Consequently, hemagglutinin‐induced ferroptosis follows a predictable pattern. In this study, hemagglutinin induces cellular ferroptosis by interacting with NCOA4 and TAX1BP1, indicating promising prospects for targeted therapy in tumors. Viruses have evolved a variety of strategies to counteract interferons, and the different viral proteins encoded by IAV achieve such aims by targeting different steps of the interferon signaling pathway. IAV PB1, PB1‐F2, NP proteins are selectively autophagic to degrade MAVS.^[^ ^29^, ^30^, ^31^ ^]^ Our results showed that hemagglutinin inhibits type I IFN by inducing lipid peroxidation, which impairs the aggregation of MAVS. Although hemagglutinin has been reported to promote viral replication by inducing the ubiquitination degradation of IFNR1 by PARP1 in previous studies,^[^ ^63^ ^]^ our data suggested that hemagglutinin promotes viral replication by a novel mechanism to inhibit MAVS activation to facilitate viral replication.

**Figure 8:**
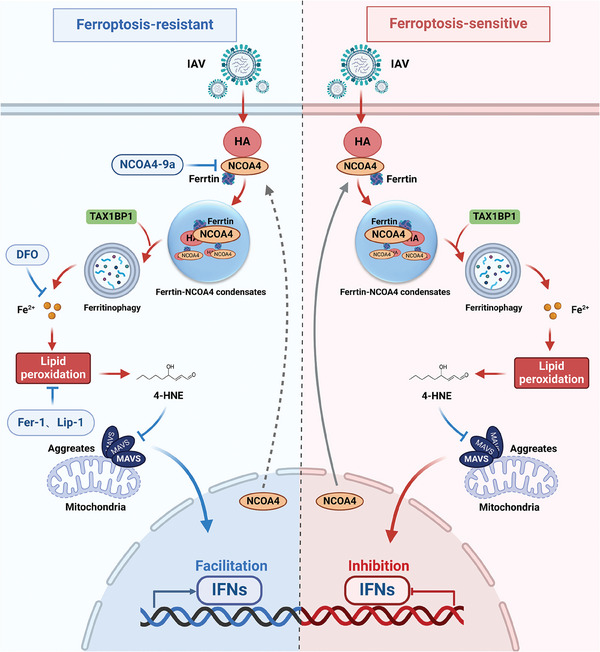
A schematic representation of IAV hemagglutinin inhibiting MAVS‐mediated antiviral immunity by inducing ferritinophagy‐induced lipid peroxidation.

In summary, our data revealed a novel mechanism by which IAV hemagglutinin modulates the MAVS‐mediated type I IFN response through ferritinophagy‐induced lipid peroxidation (**Figure** **8**). Specifically, we found that IAV hemagglutinin induces ferritinophagy by interacting with NCOA4 and TAX1BP1, which promotes the formation of ferritin‐NCOA4 condensates, thereby sensitizing cells to ferroptosis in vitro and in vivo. Significantly, we found that inhibition of ferroptosis not only suppresses IAV‐induced cell death, but also inhibits viral replication, because IAV hemagglutinin leads to lipid peroxidation by modulating ferritinophagy, which affects the abundance of lipid peroxidation products (for example, 4‐HNE), thereby impairing MAVS aggregation suppresses antiviral immunity.

## 4 Experimental Section

### Cell and Viruses

Human lung epithelial (A549, ATCC) cells, Madin‐Darby canine kidney (MDCK, ATCC) cells, and newborn pig trachea (NPTr) cells were cultured in Dulbecco's modified Eagle's medium (DMEM/HIGH GLUCOSE, HyClone USA). Human embryonic kidney (HEK293T, ATCC) cells were cultured in Roswell Park Memorial Institute‐1640 medium (RPMI 1640, HyClone). All media were supplemented with 10% fetal bovine serum (FBS, PAN‐Biotec), and the cells were cultured at 37 °C with 5% CO_2_. NPTr cells were established following the serial culture of primary cells derived from tracheal tissues, and various influenza virus subtypes isolated from human, swine, and avian species could efficiently replicate with cytopathic effects in NPTr cells.^[^ ^64^ ^]^ For NCOA4 KO cells, the cell lines were constructed using the CRISPR/Cas9 system as previously described.^[^ ^65^ ^]^ Briefly, sgRNA targeting the human NCOA4 gene (see Table S1, Supporting Information, for sequences) was cloned into the lentiCRISPR V2 vector to generate recombinant lentiviruses. NCOA4 lentiCRISPR V2 lentivirus or vector lentiCRISPR V2 lentivirus was infected in A549 cells and HEK293T cells. 48–60 h post‐transfection, puromycin (2.5 mg mL^−1^) was added to filter positive clones, and finally monoclonal cells were obtained by limited dilution. The NCOA4 KO cells were analyzed by Western blotting. The recombinant adenoviral method using the Ad5 adenoviral vector to rescue the eGFP empty vector and PR8 HA full‐length was performed as previously described,^[^ ^66^ ^]^ with a kind gift from Prof. Ling Zhao, Huazhong Agricultural University. A/Puerto Rico/8/1934 (PR8, H1N1), A/swine/Hubei/221/2016 (HuB, H1N1), and A/duck/Hubei/Hangmei01/2006 (HM, H5N1) were preserved in our laboratory.^[^ ^67^, ^68^ ^]^ Viruses were proliferated in 10‐day‐old specific‐pathogen‐free (SPF) embryonated eggs and viral titers were determined by calculating the log_10_ 50% tissue culture infectious dose (TCID_50_) mL^−1^ on MDCK cells. All experiments with the H5N1 virus were performed in a biosafety level 3 laboratory (BSL3) and were approved by the Intuitional Biosafety Committee of HZAU.

### Antibodies and Reagents

The antibodies used in this study were as follows: Rabbit anti‐NCOA4 (ab86707) was from Abcam; Mouse anti‐ARA70 (C‐4) (sc‐373739) was from SANTA CRUZ; Rabbit anti‐FTH1 (A19544), Rabbit anti‐GPX4 (A1933), Rabbit anti‐SLC7A11/xCT (A2413), Rabbit phospho‐IRF3‐S386 (AP0995), Rabbit control IgG (AC005), Horseradish peroxidase (HRP)‐conjugated goat anti‐mouse (AS003), goat anti‐rabbit (AS014), goat anti‐human (AS002), FITC goat anti‐mouse, and 647‐conjugated Goat Anti‐Mouse (AS059) were from Abclonal; Rabbit anti‐P62 (18420‐1‐AP), Rabbit anti‐Flag tag (80010‐1‐RR), Rabbit anti‐HA tag (51064‐2‐AP), Rabbit anti‐GFP tag (50430‐2‐AP), and Mouse anti‐GAPDH (60004‐1‐Ig) were from Proteintech; Rabbit anti‐MAP1LC3B/LC3B (NB100‐2220) was from Novus Biologicals. Rabbit anti‐IAV HA (GTX127294), NA (GTX125974), NP (GTX125989), NS1 (GTX125990), NS2 (GTX125953), M1 (GTX125928), and M2 (GTX125951) were from Gene Tax; FITC‐goat anti‐mouse (GM200G‐02C), FITC‐goat anti‐rabbit (GR200G‐02C), 594‐conjugated goat anti‐mouse (GM200G‐43C), 594‐conjugated goat anti‐rabbit were from SungeneBiotech. DAPI (C1005, 1:1000 dilution) and Lyso‐Tracker Red (C1046, 1:20000 dilution) was from Beyotime. The inhibitors and stimulators used in this study were as follows: RSL3 (HY‐100218A), Ferrostatin‐1 (Fer‐1, HY‐100579), Liproxstatin‐1 (Lip‐1, HY‐12726), Deferoxamine mesylate (DFO, HY‐B0988), Chloroquine (CQ, HY‐17589A), and Polyinosinic‐polycytidylic acid (Poly (I:C), HY‐107202) were MCE; NCOA4‐9a (2650557‐72‐3), and 4‐Hydroxynonenal (4‐HNE, 75899‐68‐2) were from TargetMol.

### Plasmids and Small Interfering RNA Oligonucleotides

The plasmids used in this study are as follows: The gene encoding PR8 hemagglutinin (HA) with HA tag or Flag tag was cloned into the pcDNA3.4 vector; The gene encoding human NCOA4 was cloned into HA‐pCAGGS, p3xFlag‐CMV and pEGFP‐N1 vectors; The gene encoding human FTH1 was cloned into the eGFP‐N1 vector. The PHW2000 plasmid encoding the HA, NA, NP, NS, and M gene fragments of the PR8 H1N1 virus, and the HA‐pCAGGS, p3xFlag‐CMV, pEGFP‐N1, IFNβ‐luc and RL‐TK vectors were maintained in the laboratory.^[^ ^28^, ^69^ ^]^ The above plasmids were transfected into HEK293T cells using Lipo8000 (Beyotime), and subsequent experiments were performed 24 h post‐transfection. A small interfering RNA (siRNA) was transfected into A549 or HEK293T cells by Lipofectamine 2000 (Invitrogen), and subsequent experiments were performed after 24 h. The primers used to construct the plasmids and siRNA are presented in Tables S1 and S3 (Supporting Information), respectively.

### Transfection and Infection

For transfection, HEK293T cells were cultured to 80% confluence and then transfected with plasmids or siRNAs using Liposome 8000 (Beyotime) or Liposome 2000 (Invitrogen) following the manufacturer's instructions. For infection, A549 or MDCK cells were first washed with phosphate buffer solution (PBS) and then exposed to PR8 H1N1 or HM H5N1 at a multiplicity of infection (MOI) of 0.01 for 1 h. Cells were resuspended in DMEM containing 0.25 µg mL^−1^ of TPCK‐treated trypsin (Sigma) and collected at the specified time points after infection. A549 cells were exposed to recombinant adenovirus Ad5‐eGFP or Ad5‐HA_PR8_ at a MOI of 0.1, and cell supernatants were discarded. Cells were collected at 48 hpi. All experiments involving viral infections were conducted in biological safety cabinets.

### Mouse Infection

Two groups of 6–8 weeks old female BALB/c mice (*n* = 5) were lightly anesthetized and intranasally inoculated with 10^8^ TCID_50_ mL^−1^ recombinant adenovirus Ad5‐eGFP or Ad5‐HA_PR8_ (50 µL). After 3 days of infection, the mice were euthanized, and lung tissues were collected for western blotting, flow cytometry, indirect immunofluorescence, and other analyses. The experimental mice were obtained from the Laboratory Animal Center of Huazhong Agricultural University (HAZU). This study was conducted in strict accordance with the recommendations provided in the Guide for the Care and Use of Laboratory Animals of the Ministry of Science and Technology of the People's Republic of China. Animal experiments were approved by the Hubei Administrative Committee for Laboratory Animals (HZAUMO‐2024‐0168).

### Virus Titration

For IAV growth kinetics analysis, viral supernatants collected at the specified time points were titrated on MDCK cells. Briefly, the serially diluted supernatants were inoculated onto MDCK cell monolayers in 96‐well plates for 1 h, after which the inoculum was removed and replaced with DMEM containing 0.25 µg mL^−1^ Trypsin‐TPCK. The plates were incubated for 72 h at 37 °C. Virus titers were determined by calculating the TCID_50_/mL using the Reed‐Muench method.

### Cell Viability and Cell Death Assay

The cell viability was determined using a cell counting kit (CCK‐8, GlpBio) according to the instructions of the manufacturer. Briefly, 1 × 10^4^ cells per well were cultured in a 96‐well plate, and at the end of the different treatments, 10 µl of CCK‐8 was added to each well and incubated at 37 °C for 1 h. The absorbance was measured at 450 nm using a microplate reader (PerkinElmer). Cell death was analyzed with Propidium Iodide (PI, Beyotime) staining and bright‐field (BF) cells by fluorescence microscopy. A total of 1 × 10^5^ cells were seeded in confocal dishes, after cells grew to full monolayer, infected with PR8 H1N1 (MOI = 0.1) for 24 h, stained with PI for 1 h, fixed in 4% paraformaldehyde and imaged by fluorescence microscopy.

### Intracellular Glutathione (GSH) and Malondialdehyde (MDA) Measurement

The intracellular GSH levels were measured by GSH and GSSG assay kit (Beyoyime). Cells were cultured in six‐well plates and after different treatments, the samples were subjected to two rapid freeze‐thaws using liquid nitrogen and a 37 °C water bath. The supernatants were centrifuged and used for GSH measurements. According to the manufacturer's instructions, the supernatant to be tested, GSH clear auxiliary solution, and total GSH assay working solution were added sequentially. The reaction was carried out at 25 °C for 1 h. NADPH (0.5 mg mL^−1^) was added and the reaction was carried out at 25 °C for 25 min, and the absorbance at 405 nm was measured using a microplate reader to calculate the GSH concentration. The lipid peroxidation levels were measured using a colorimetric reaction based on the reaction of MDA and thiobarbituric acid (TBA) to produce a red product. Lipid Peroxidation MDA assay kit (Beyotime) instructions were followed exactly, cell lysates from different treatments or adenovirus‐infected lung tissue homogenates were mixed with MDA assay solution, heated at 100 °C for 15 min, cooled to room temperature, and centrifuged at 1000 × *g* for 10 min. The supernatant was then collected, and absorbance was measured at 450 nm using a microplate reader.

### Intracellular Iron Measurement

The intracellular or lung tissue iron concentration was measured using the Total Iron Content Colorimetric Assay Kit (Applygen). Cell samples were processed after differential treatments according to the manufacturer's instructions, and the iron concentration was calculated by measuring the absorbance at nm using a microplate reader. The protein concentration in the sample lysate was determined by the BCA method to correct for the iron concentration in the samples. The ferrous iron levels of the unstable intracellular iron pool (LIP) were determined by the FerroOrange (Dojindo) probe. For flow cytometry (Beckman Coulter), 1 × 10^5^ cells with differential treatments or adenovirus‐infected lung tissue cells were collected and stained for 30 min at 37 °C using a FerroOrange probe (1 µm), and fluorescence intensity was measured directly. For confocal microscopy, the different treated cells were incubated with the FerroOrang probe at 37 °C for 30 min, and the images were presented by confocal microscopy (NIKON N‐SIM).

### ROS and Lipid Peroxidation Levels Measurement

For the cellular total ROS level assay, cells were incubated with PBS solution containing 5 µm H2DCFDA probe (MCE) at 37 °C for 30 min in light‐avoidance after different treatments, then cells were collected with 0.05% trypsin‐EDTA solution, suspended in fresh medium and immediately analyzed by flow cytometry. For lipid peroxidation level assay, differently treated cells or adenovirus‐infected lung tissue cells were added with C11 BODIPY 581/ 591 (10 µm, Abclonal) and incubated for 1 h at 37 °C. Lipid peroxidation levels were analyzed by flow cytometry and confocal microscopy.

### Immunofluorescence Assay (IFA)

HEK293T or A549 cells were seeded into confocal dishes and transfected with the indicated plasmids or infected with the indicated IAV. 4% paraformaldehyde was used to fix the cells or paraffin‐embedded lung tissue sections for 10 min, 0.1% Triton X‐100 was used to permeabilize the cells for 15 min, and then the cells were blocked with PBS containing 5% goat serum for 1 h. Subsequently, the cells were incubated with the primary antibody for 2 h, and incubated with the corresponding fluorescent secondary antibody for 1 h, DAPI staining for 10 min, and the final images were visualized by confocal micrography.

### Coimmunoprecipitation and Western Blotting

Cells or lung tissue homogenates were lysed in western blotting and IP (Beyotime) or mammalian cell lysis buffer (CWBIO) with protease inhibitors (Biosharp) for 30 min at 4 °C. Subsequently separated by SDS‐PAGE or SDD‐AGE, the samples were transferred to nitrocellulose membranes, which were blocked with 5% skimmed milk (BD Life Sciences) for 1 h, incubated with the corresponding primary antibody for 2 h, and HRP‐conjugated with the corresponding secondary antibody for 1 h. The semidenaturing detergent agarose gel electrophoresis (SDD‐AGE) was carried out as described previously.^[^ ^44^ ^]^ Briefly, cell lysates were resuspended in 1× sample buffer (0.5 × TBE (Servicebio), 10% glycerol (Sigma), 2% SDS (Sigma), and 0.0025% bromophenol blue (Sigma)), were added to a vertical 1.5% agarose gel, electrophoresed in running buffer (1× TBE and 0.1% SDS) at a constant voltage of 100 V for 1 h at 4 °C, and subsequently transferred to nitrocellulose membranes for western blotting analysis. For Co‐IP assay, 30 µL of Protein A/G Magnetic Beads (MCE) were added with 2–3 g of the indicated primary antibodies and incubated at 4 °C for 8 h, supernatant was removed. The magnetic beads with conjugated antibodies were co‐incubated with cell lysate at 4 °C for 8–10 h. The lysate was removed and the magnetic beads were used for western blotting analysis. Images were obtained by the ECL detection system (Tanon‐5200; Tano) and quantified with ImageJ software.

### LC–MS/MS Analysis

HEK293T cells were transfected with vector or Flag‐HA_PR8_, and cell lysates were harvested at 36 h. Proteins in the lysates were enriched using rabbit‐conjugated anti‐Flag magnetic beads for mass spectrometry analysis. The IP samples were enzymatically digested and desalted according to published methods.^[^ ^70^ ^]^ Briefly, the separation was performed using a Nano‐HPLC liquid phase system UltiMate 3000 RSLCnano (ThermoFisher Scientific). The samples were loaded by an autosampler and combined on a Trap column, then separated on an Analysis column, 75 µm × 150 mm (RP‐C18, New Objective, USA) at a flow rate of 300 nL min^−1^. The samples were cleaned up once between samples with a 30 min mobile‐phase gradient of blank solvent. The enzymatic products were separated by capillary high‐performance liquid chromatography (HPLC) and analyzed by mass spectrometry using a Q‐Exactive plus mass spectrometer (ThermoFisher Scientific). Detection mode: calibrated by standard calibration solution before use, parent ion scanning range: 300–1500 *m*/*z*, mass spectrometry scanning mode under the information‐dependent acquisition working mode (DDA, Data Dependent Acquisition), the strongest 20 fragmentation patterns were collected after each full scan (MS2 scan), fragmentation mode: HCD (high energy collision dissociation), NCE energy 28, dynamic exclusion time: 25 s. MS1 resolution at M/Z 200 was 70 000, AGC target was set to 3e6, and the maximum injection time was 100 ms, and MS2 resolution was set to 17 500, AGC target was set to 1e5, maximum injection time 50 ms, MS2 resolution was set to 17 500, AGC target was set to 1e5, maximum injection time 50 ms. The retrieved proteins were analyzed by GO/KEGG through the OebiotechCloud.

### Luciferase Activity Assay

The luciferase activity of IFNβ was determined using a Dual‐Luciferase Reporter Assay system (Promega), according to the manufacturer's instructions. The transfection efficiency was normalized by the ratio of the firefly luciferase activity to the *Renilla* activity. HEK293T cells were cultured in 12‐well plates and transfected with 0.02 µg RL‐TK and 0.3 µg IFNβ‐luc per well, as well as MVAS or poly (I:C). Followed by NCOA4 KO or treatment with different inhibitors, and cell lysates were harvested 24–36 h and luciferase activity was analyzed.

### Quantitative real‐Time PCR (qRT‐PCR) Assay

The cells were infected with PR8 H1N1 virus (MOI = 0.1) for 24 h or Poly (I:C) transfection for 12 h in NCOA4 KO or WT A549 cells, and cell samples were harvested. For HEK293T cells, after Poly (I:C) transfection for 6 h, Flag‐HA_PR8_ plasmid was transfected and cell samples were harvested at 24 h. Total RNA from the above cells were extracted by the RNAiso Plus kit (Takara) with DNase I, the concentration of extracted RNA was measured by NanoDrop 2000 spectrophotometer (Thermo Fisher Scientific) and the integrity of the RNA was evaluated by 1% agarose gel electrophoresis. Equal amounts of total RNA were passed through a reverse transcription kit (Vazyme) for cDNA synthesis. Primers used for qRT–PCR of this experiment are given in Table S2 (Supporting Information). Each sample was tested in triplicate. The specificity of the amplified target gene was assessed using dissociation curve analysis. The target gene relative expression levels vs the β‐actin gene (selected as the reference gene) was calculated according to the 2^−ΔΔCT^ method.^[^ ^71^ ^]^ To determine the relative fold change of the target gene at different time points, the expression value was normalized using the corresponding control group.

### Statistical Analysis

In this study, statistical analysis and presentation graphics were carried out by the GraphPad Prism 8.0 software. Results were shown as mean ± SEM from at least three independent experiments, and statistical significance was analyzed by a two‐tailed Student t‐test. The *p* values< 0.05 are deemed statistically significant difference (*p < 0.05; **p < 0.01; ***p < 0.01; ns, no significant).

## Conflict of Interest

The authors declare no conflict of interest.

## Supporting information

Supporting Information

## Data Availability

The data that support the findings of this study are available in the supplementary material of this article.
