# Supplementary material for: The Hemagglutinin of Influenza A Virus Induces Ferroptosis to Facilitate Viral Replication
Source: Adv Sci (Weinh). 2024 Aug 19;11(39):2404365. doi: 10.1002/advs.202404365 (PMC11497066; doi:10.1002/advs.202404365)
Supplement: Supplementary file 1 — Supporting Information [file ADVS-11-2404365-s001.docx]

# Supporting information

The Hemagglutinin of Influenza A Virus Induces Ferroptosis to Facilitate Viral Replication

Aotian Ouyang, Tong Chen, Yi Feng, Jiahui Zou, Shaoyu Tu, Meijun Jiang, Huimin Sun, Hongbo Zhou*


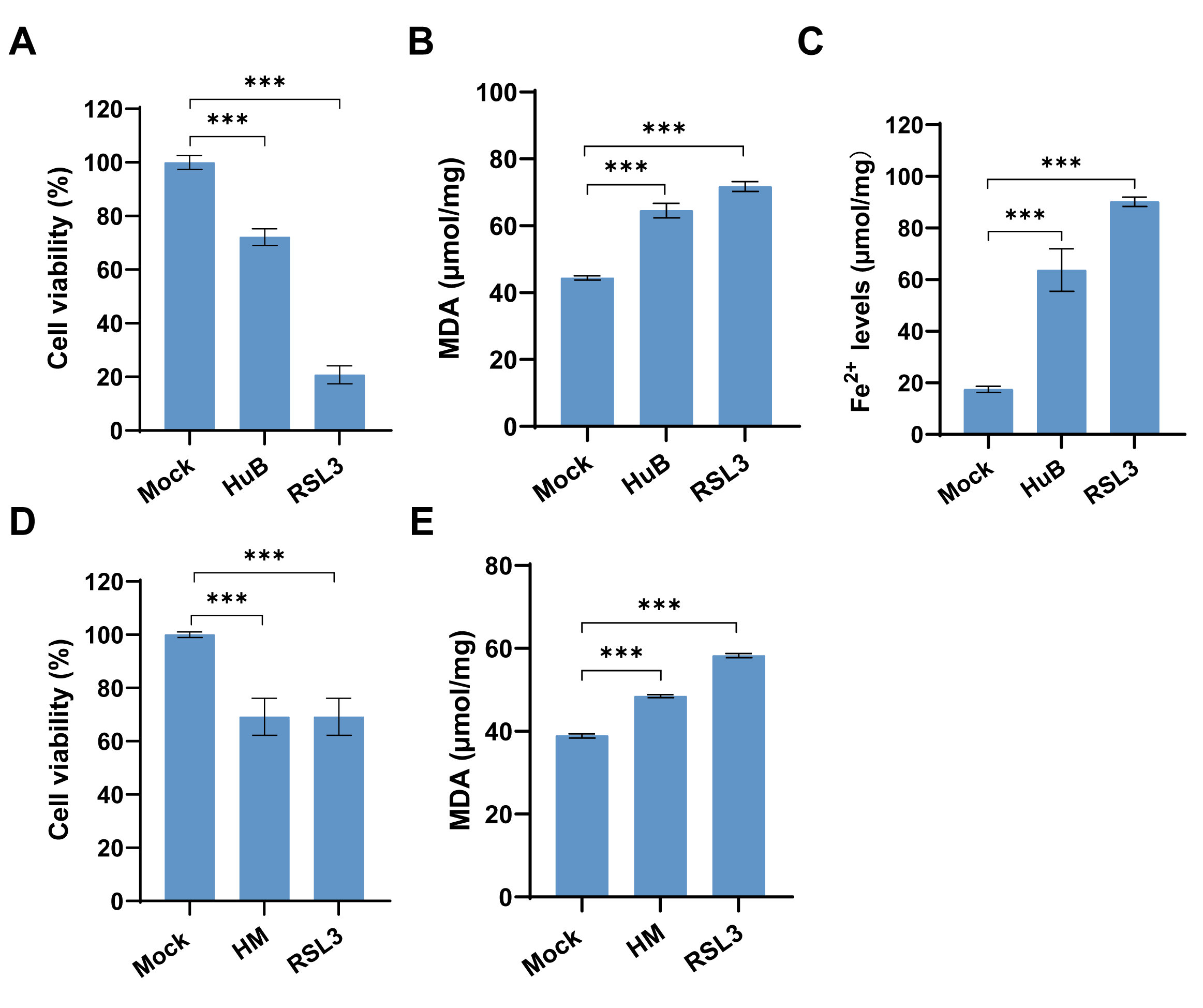
 **Figure S1. Cellular ferroptosis is triggered by HuB H1N1 and HM H5N1.**

Cells were treated with RSL3 (2 μM) or infected with IAV (MOI = 0.1) for 24 hpi, respectively. (A) The cell viability of HuB H1N1 virus-infected NPTr cells was detected by CCK-8 Kit. (B) MDA concentration in HuB H1N1 virus-infected NPTr cells was measured by MDA assay kit. (C) The Fe^2+^ concentration in HuB-infected NPTr cells was detected by the iron assay kit. (D) The cell viability of HM H5N1virus-infected A549 cells was detected by CCK-8 kit. (E) MDA concentration in HM H5N1 virus-infected A549 cells was measured by MDA assay kit. Data were shown as means ± SEM (*n* = 3) from triplicate independent experiments, and significance was analyzed by two-tailed Student's t-test. (****p* < 0.001).


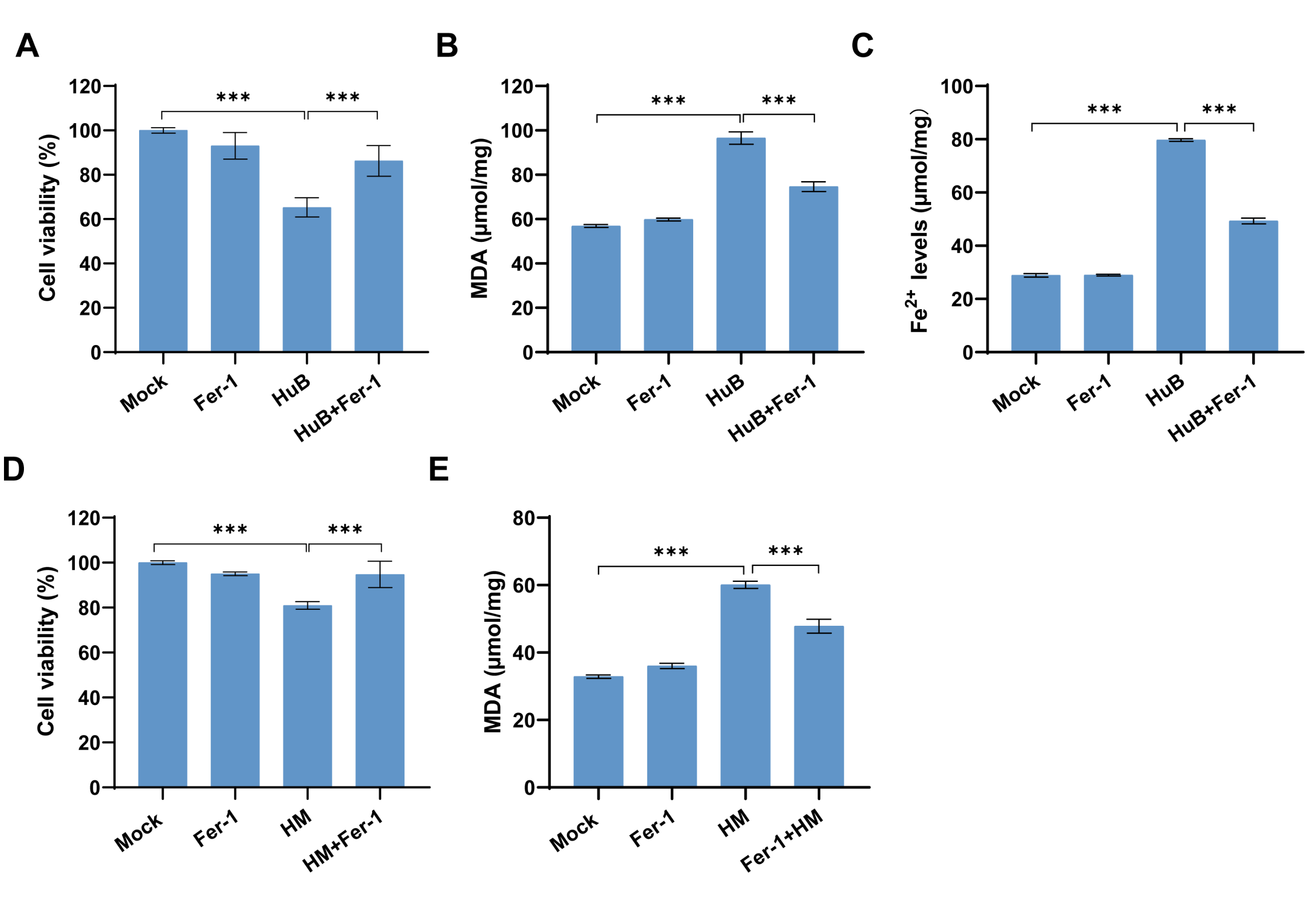


**Figure S2. Fer-1 inhibits cellular ferroptosis induced by HuB H1N1 and HM H5N1.**

Cells were pretreated with Fer-1 (5 μM) or vehicle (DMSO) for 2 hpi, followed by infected cells with IAV (MOI = 0.1) in Fer-1 (5 μM) or vehicle (DMSO) treatment. (A) The cell viability of HuB H1N1 virus-infected NPTr cells was detected by CCK-8 kit. (B) MDA concentration in HuB H1N1 virus-infected NPTr cells was measured by MDA assay kit. (C) The Fe^2+^ concentration in HuB-infected NPTr cells was detected by the iron assay kit. (D) The cell viability of HM H5N1 virus-infected A549 cells was detected by CCK-8 Kit. (E) MDA concentration in HM H5N1 virus-infected A549 cells was measured by MDA assay kit. Data were shown as means ± SEM (*n* = 3) from triplicate independent experiments, and significance was analyzed by two-tailed Student's t-test. (****p* < 0.001).


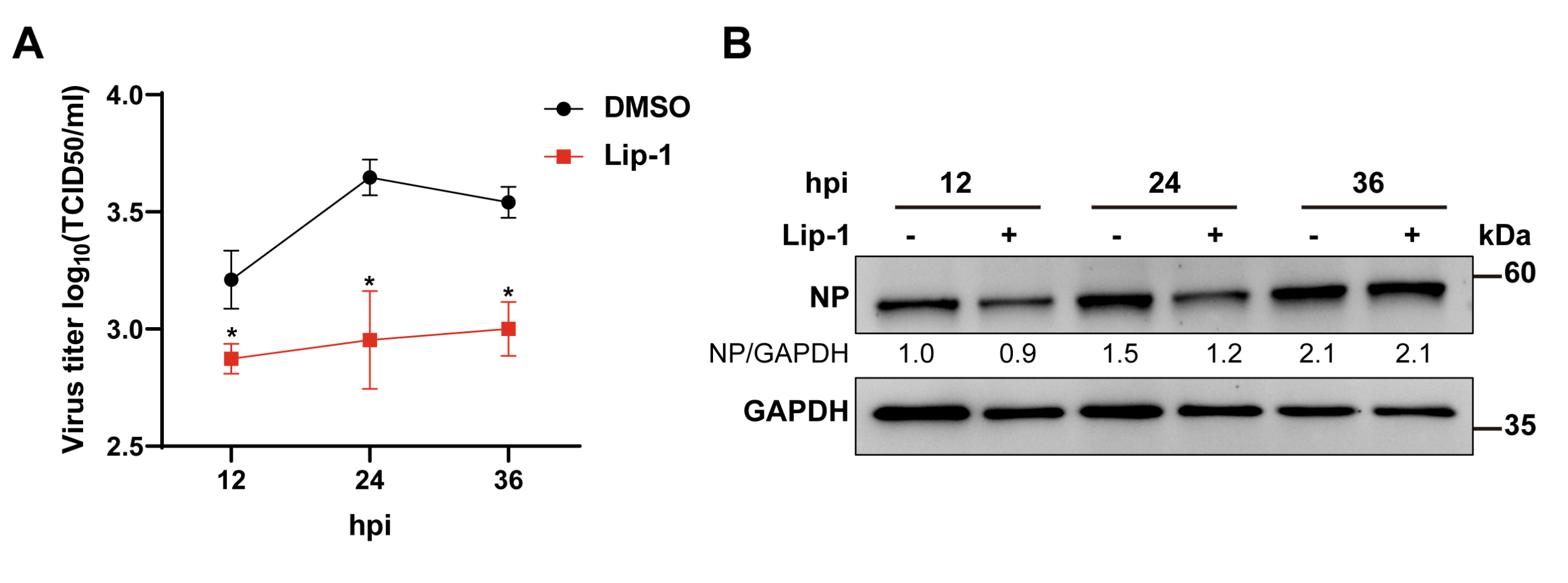


**Figure S3. Lip-1 inhibits IAV replication.**

A549 cells were pretreated with Lip-1 (100 μM) or vehicle (DMSO) for 2 hpi, followed by infected A549 cells with PR8 H1N1 virus (MOI = 0.1) in Lip-1 (100 μM) or vehicle (DMSO) treatment, and cell supernatants and lysates were harvested at 12, 24, and 36 hpi, respectively. (A) Viral titers in MDCK cell supernatants were determined by TCID_50_. (B)The viral protein NP expression detected by western blotting. Data were shown as means ± SEM (*n* = 3) from triplicate independent experiments, and significance was analyzed by two-tailed Student's t-test. (**p* < 0.05).


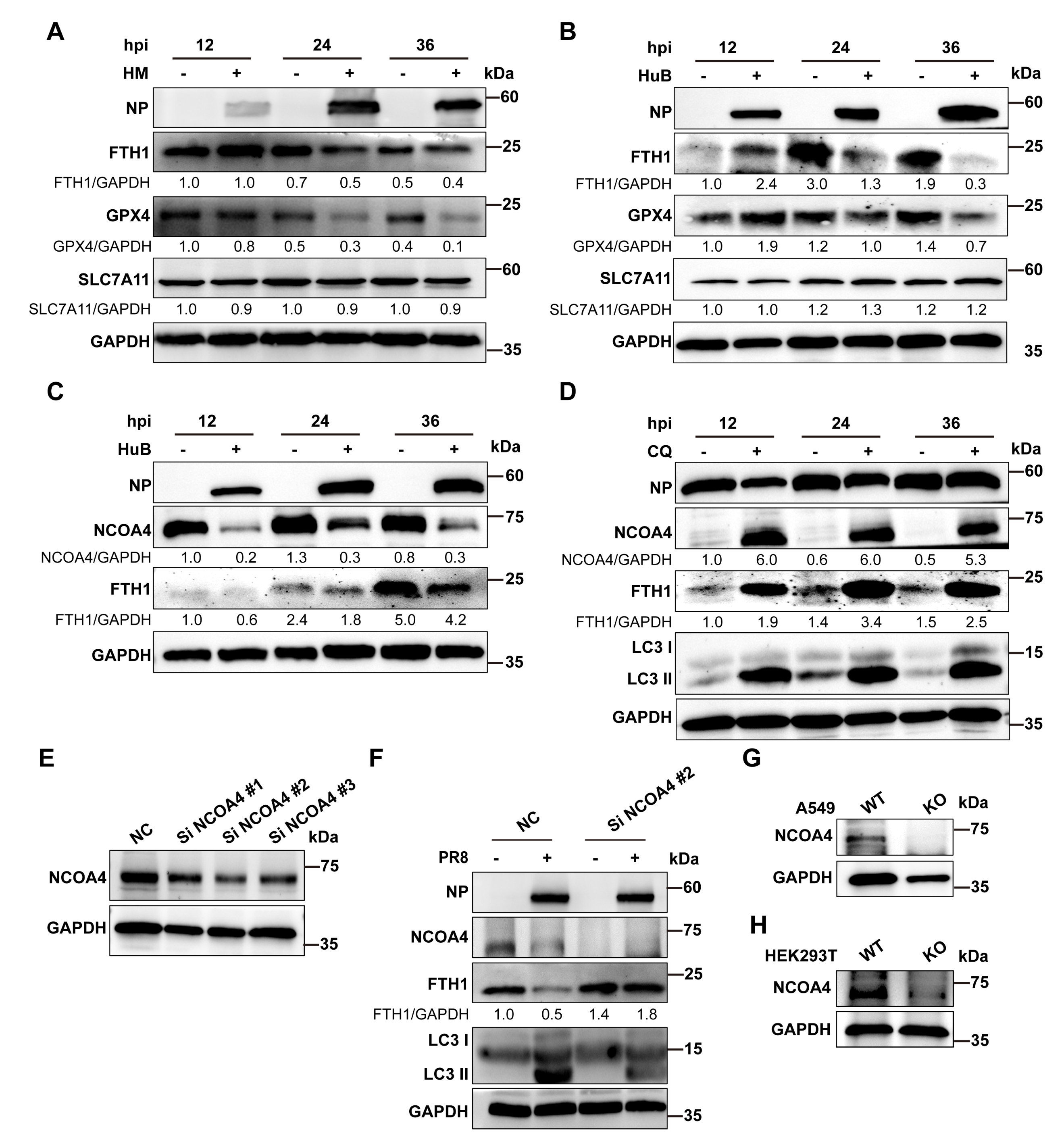


**Figure S4. IAV triggers cellular ferroptosis by inducing ferritinophagy.**

(A-C) Cells were infected with IAV (MOI = 0.1), and cell lysates were harvested at 12, 24, and 36 hpi. (A) The protein expression levels of ferroptosis in HM H5N1 virus-infected A549 cells was analyzed by western blotting. (B) The protein expression levels of ferroptosis in HuB H1N1 virus-infected NPTr cells was analyzed by western blotting. (C) The protein expression levels of ferritinophagy in HuB H1N1 virus-infected NPTr cells was analyzed by western blotting. (D) The protein expression of ferritinophagy was analyzed by western blotting for HuB H1N1 virus infection treated with CQ. NPTr cells infected with HuB H1N1 virus (MOI = 0.1) at the treatment of CQ (100 μM) and cell lysates were harvested at 12, 24, and 36 hpi for western blotting analysis. (E) The knockdown efficiency of Si NCOA4 was detected by western blotting. A549 cells were transfected with Si NC and Si NCOA4, respectively, and the cell lysates were harvested at 24 h and the protein expression of NCOA4 was detected by western blotting. (B) The protein expression of ferritinophagy genes and viral nucleoprotein following PR8 H1N1 virus infection in knockdown NCOA4 cells. A549 cells were transfected with Si NC or Si NCOA4 for 24 h and infected with PR8 H1N1 virus (MOI = 0.1) for 24 hpi, Cell lysates were harvested for western blotting analysis. (G) The efficiency of NCOA4 KO in A549 cells was analyzed by western blotting. (H) The efficiency of NCOA4 KO in HEK293T cells was analyzed by western blotting. Data were shown as means ± SEM (*n* = 3) from triplicate independent experiments, and significance was analyzed by two-tailed Student's t-test.


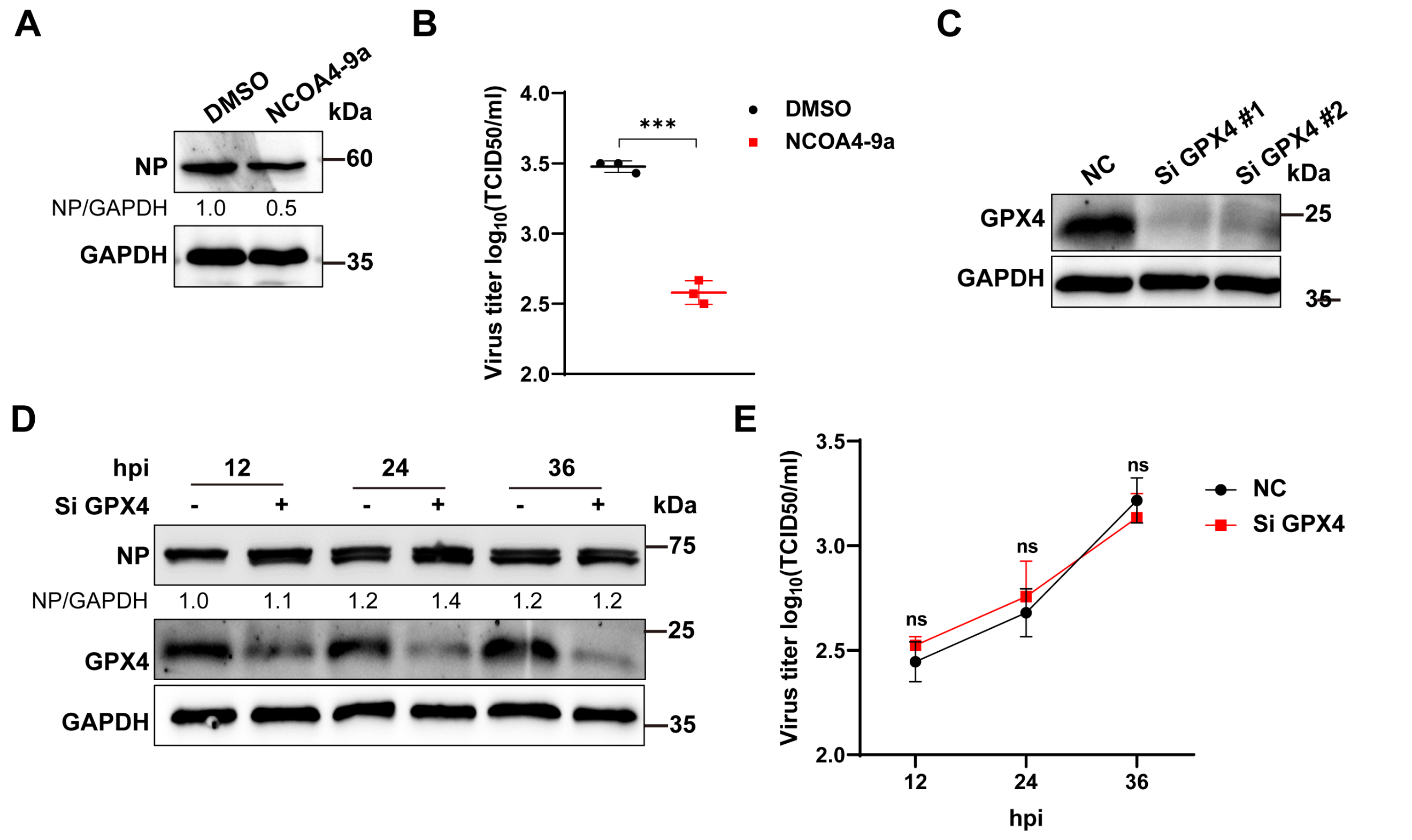


**Figure S5. Inhibition of NCOA4-mediated ferritinophagy suppresses IAV replication.**

(A-B) A549 cells were pretreated with NCOA4-9a (2.5 μM) or vehicle (DMSO) for 2 hpi, followed by infected A549 cells with PR8 H1N1 virus (MOI = 0.1) in NCOA4-9a (2.5 μM) or vehicle (DMSO) treatment, and cell supernatants and lysates were harvested at 24 hpi. (A) The viral protein NP expression detected by western blotting. (B) Viral titers in MDCK cell supernatants were determined by TCID_50_. (C) The knockdown efficiency of GPX4 in A549 cells was analyzed by western blotting. A549 cells were transfected with Si NC or Si GPX4 for 24 h, and cell lysates were harvested for western blotting analysis. (D-E) The Si NC or Si GPX4 were transfected in A549 cells for 24 h, followed by infection with PR8 H1N1 virus (MOI = 0.1), and cell supernatants and lysates were harvested at 12, 24, and 36 hpi. (D) The viral protein NP expression detected by western blotting. (E) Viral titers in MDCK cell supernatants were determined by TCID_50_. Data were shown as means ± SEM (*n* = 3) from triplicate independent experiments, and significance was analyzed by two-tailed Student's t-test. (****p* < 0.001; ns, no significant).


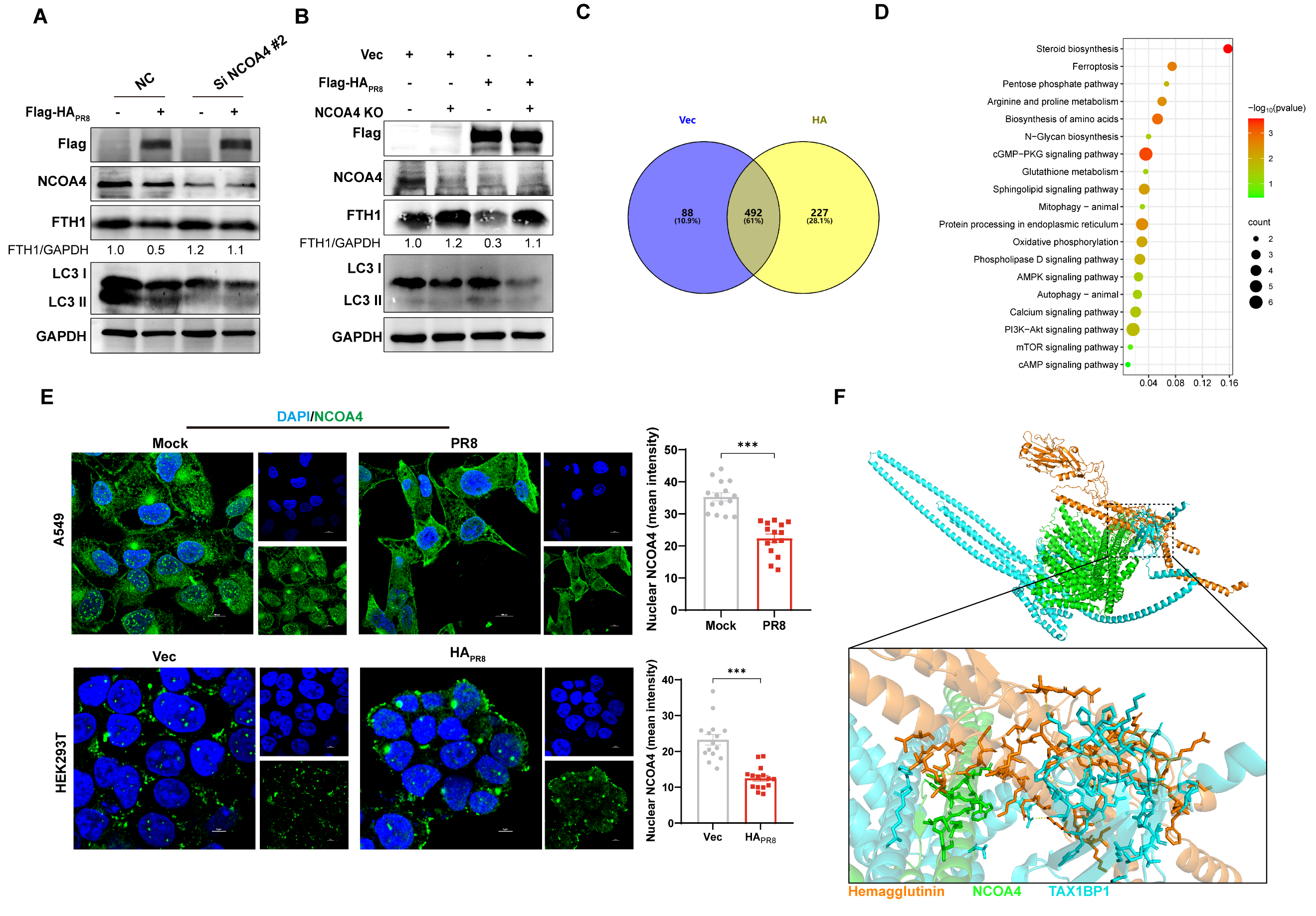


**Figure S6. IAV hemagglutinin induces NCOA4-mediated ferritinophagy.**

(A) The effect of overexpression of PR8 HA to protein expression of ferritinophagy in NCOA4 knockdown cells. HEK293T cells were transfected with Si NC or Si NCOA4, transfected with vector or Flag-HA_PR8_ at 24 h, and cell lysates were harvested after 24 h for western blotting analysis. (B) The effect of overexpression of PR8 HA to protein expression of ferritinophagy in NCOA4 KO cells. HEK293T WT and NCOA4 KO cells were transfected with vector or Flag-HA_PR8_, the cell lysates were harvested at 24 h after transfection for western blotting analysis. (C) A venn diagram showing the number of differentially interacting proteins identified by LC-MS/MS in HEK293T cells transfected with vector or Flag-HA_PR8_. (D) KEGG pathway enrichment analysis of Flag-HA_PR8_-interacting proteins. (E) The subcellular localization of NCOA4 in PR8 H1N1 virus-infected A549 cells or PR8 HA-transfected HEK293T cells. PR8 H1N1 virus (MOI = 0.1) infected A549 cells for 24 hpi. Quantification of the nuclear localization intensity of NCOA4 was analyzed for 15 cells in different visual fields. HEK293T cells were transfected with vector or HA_PR8_. Scale bar = 100 px. Corresponding antibody indicated to NCOA4 (Green), DAPI indicated to nucleus. Scale bar = 5 μm. (F) The interaction structure between PR8 hemagglutinin, NCOA4 and TAX1BP1 was predicted by AlphaFold3 and visualized by Pymol. Data were shown as means ± SEM (*n* = 3) from triplicate independent experiments, and significance was analyzed by two-tailed Student's t-test. (****p* < 0.001).


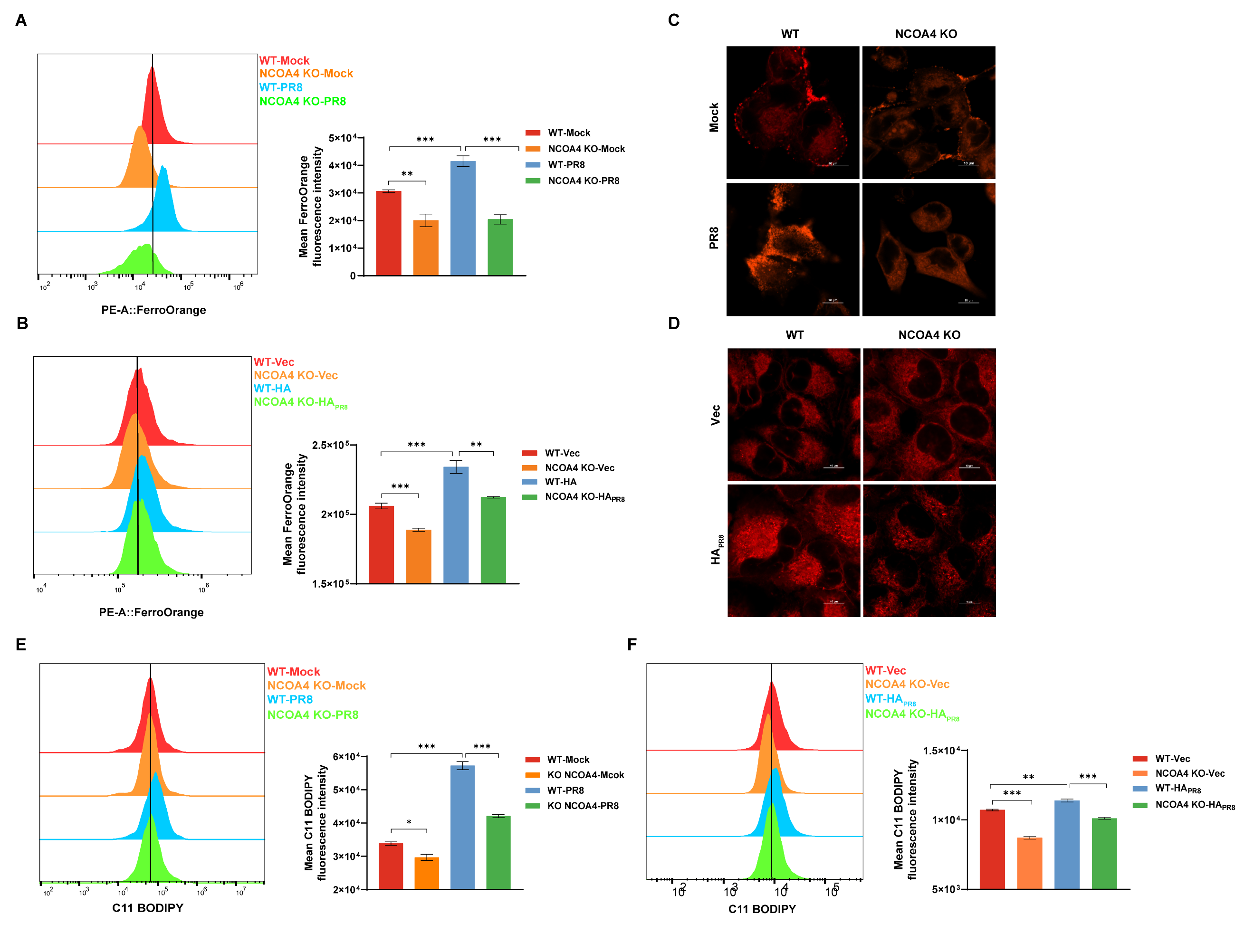


**Figure S7.** **IAV hemagglutinin impacts lipid peroxidation levels via ferritinophagy.**

(A-D) PR8 H1N1 virus (MOI = 0.1) infected or PR8 HA-transfected WT or NCOA4 knockout KO cells, the FerroOrange probe labeled cells with destabilized ferrous iron and was analyzed. (A-B) Flow cytometry analysis of destabilized ferrous iron levels with PR8 H1N1 virus infection or PR8 HA transfection in WT or NCOA4 KO cells. (C-D) Confocal analysis of destabilized ferrous iron levels after PR8 H1N1 virus infection or PR8 HA transfection in WT or NCOA4 KO cells. (E-F) The lipid ROS levels in PR8 H1N1 virus-infected or PR8 HA-transfected WT or NCOA4 KO cells were analyzed by flow cytometry. PR8 H1N1 virus (MOI = 0.1) infected or PR8 HA-transfected WT or NCOA4 KO cells were subsequently analyzed by flow cytometry for C11 BODIPY probe labeled lipid ROS levels. Data were shown as means ± SEM (*n* = 3) from triplicate independent experiments, and significance was analyzed by two-tailed Student's t-test. (**p* < 0.05; ***p* < 0.01; ****p* < 0.001).

**Table S1.**

**Primers used in this study for PCR.**

| Primer name | Sequence (5'–3') |
| --- | --- |
| HA-HA_PR8_ | F: CGACGCGTaaATGAAGGCAAACCTACTGG |
|  | R: AGCTTTGTTTAAACtGGCGTAATCTGGGACGTCGTATGGGTAGGTGG  CGATGCATATTCTGCACTGCA |
| Flag-HA_PR8_ | F: CCCAAGCTTATGAAGGCAAACCTACTGGTCC |
|  | R: CCGGATATCCTTGTCATCGTCATCCTTGTAGTCGATGTCATGATCTT  TATAATCACCGTCATGGTCTTTGTAGTCGATGCATATTCTGCACTGCA |
| HA-NCOA4 | F: CCGGAATTCATGAATACCTTCCAAGACCAGAGTGG |
|  | R: CCGCTCGAGCATCTGTAGAGGAGTTCGATATAACCAC |
| Flag-NCOA4 | F: CCGGAATTCaATGAATACCTTCCAAGACCAGAGTGG |
|  | R: CGCGGATCCCATCTGTAGAGGAGTTCGATATAACCAC |
| GFP-NCOA4 | F: CCGGAATTCaaATGAATACCTTCCAAGACCAGAGTGG |
|  | R: CGCGGATCCaaCATCTGTAGAGGAGTTCGATATAACCAC |
| GFP-FTH1 | F: CCGGAATTCaaATGACGACCGCGTCCAC |
|  | R: CGCGGATCCaaGCTTTCATTATCACTGTCTCCCAGG |
| NCOA4-sgRNA | F: CACCgTGAGGTGTAGTGATGCACGG |
|  | R: AAACCCGTGCATCACTACACCTCAC |

**Table S2.**

**Primers used in this study for qRT-PCR.**

| Primer name | Sequence (5'–3') |
| --- | --- |
| IFNβ | F: CCTCATTGCTACTGCCCTCT |
|  | R: GGTGTGGTGTCCGAGGAATA |
| GAPDH | F: GCAAAGGCTGTGGGCAAGG |
|  | R: GGAGGAGTGGGTGTCGCTG |

**Table S3.**

**RNA oligonucleotides were used in this study.**

| **Primer name** | **Primer sequence** |
| --- | --- |
| NC | F: UUCUCCGAACGUGUCACGUTT |
|  | R: AAGAGGCUUGCACAGUGCA |
| Si NCOA4#1 | F: GGACCUUAUUUAUCAGCUUTT |
|  | R: AAGCUGAUAAAUAAGGUCCTT |
| Si NCOA4#2 | R: GCUGUCCCUUUCAGCGAAUTT |
|  | F: AUUCGCUGAAAGGGACAGCTT |
| Si NCOA4#3 | F: GCAUAAAGAUUCCCUGAAUTT |
|  | R: AUUCAGGGAAUCUUUAUGCTT |
| Si GPX4#1 | F: GGAGUAACGAAGAGAUCAA |
|  | R: UUGAUCUCUUCGUUACUCC |
| Si GPX4#2 | F: GCAAGACCGAAGUAAACUA |
|  | R: UAGUUUACUUCGGUCUUGC |

**Data S1. (separate file)**

H1N1 hemagglutmin interactions protein profile.
